# Introgressing cry1Ac for Pod Borer Resistance in Chickpea Through Marker-Assisted Backcross Breeding

**DOI:** 10.3389/fgene.2022.847647

**Published:** 2022-04-12

**Authors:** Ajinder Kaur, Urvashi Sharma, Sarvjeet Singh, Ravinder Singh, Yogesh Vikal, Satnam Singh, Palvi Malik, Khushpreet Kaur, Inderjit Singh, Shayla Bindra, Bidyut Kumar Sarmah, Jagdeep Singh Sandhu

**Affiliations:** ^1^ School of Agricultural Biotechnology, Punjab Agricultural University, Ludhiana, India; ^2^ Pulses Section, Department of Plant Breeding and Genetics, Punjab Agricultural University, Ludhiana, India; ^3^ Punjab Agricultural University, Regional Research Station, Faridkot, India; ^4^ Department of Biotechnology-Assam Agricultural University Centre, Assam Agricultural University, Jorhat, India

**Keywords:** Cicer arietinum, Mendelian inheritance, transgene introgression, Helicoverpa armigera resistance, marker-assisted backcross breeding

## Abstract

The gram pod borer *Helicoverpa armigera* is a major constraint to chickpea (*Cicer arietinum* L.) production worldwide, reducing crop yield by up to 90%. The constraint is difficult to overcome as chickpea germplasm including wild species either lacks pod borer resistance or if possessing resistance is cross-incompatible. This study describes conversion of elite but pod borer-susceptible commercial chickpea cultivars into resistant cultivars through introgression of cry1Ac using marker-assisted backcross breeding. The chickpea cultivars (PBG7 and L552) were crossed with pod borer-resistant transgenic lines (BS 100B and BS 100E) carrying cry1Ac that led to the development of BC_1_F_1_, BC_1_F_2_, BC_1_F_3_, BC_2_F_1_, BC_2_F_2_, and BC_2_F_3_ populations from three cross combinations. The foreground selection revealed that 35.38% BC_1_F_1_ and 8.4% BC_1_F_2_ plants obtained from Cross A (PBG7 × BS 100B), 50% BC_1_F_1_ and 76.5% BC_1_F_2_ plants from Cross B (L552 × BS 100E), and 12.05% BC_2_F_2_ and 82.81% (average) BC_2_F_3_ plants derived from Cross C (PBG7 × BS 100E) carried the cry1Ac gene. The bioassay of backcross populations for toxicity to *H*. *armigera* displayed up to 100% larval mortality. BC_1_F_1_ and BC_1_F_2_ populations derived from Cross B and BC_2_F_3_ population from Cross C segregated in the Mendelian ratio for cry1Ac confirmed inheritance of a single copy of transgene, whereas BC_1_F_1_ and BC_1_F_2_ populations obtained from Cross A and BC_2_F_2_ population from Cross C exhibited distorted segregation ratios. BC_1_F_1_ plants of Cross A and Cross B accumulated Cry1Ac protein ranging from 11.03 to 11.71 µgg^−1^ in leaf tissue. Cry1Ac-positive BC_2_F_2_ plants from Cross C demonstrated high recurrent parent genome recovery (91.3%) through background selection using SSR markers and phenome recovery of 90.94%, amongst these 30% plants, were homozygous for transgene. The performance of BC_2_F_3_ progenies derived from homozygous plants was similar to that of the recurrent parent for main agronomic traits, such as number of pods and seed yield per plant. These progenies are a valuable source for *H*. *armigera* resistance in chickpea breeding programs.

## Introduction

Chickpea (*Cicer arietinum* L., *2n* = 16), belonging to the family Leguminoseae, is an economical source of protein (18–22%), minerals, fiber, β-carotene, and unsaturated fatty acids (Jukanti et al., 2012). The crop is grown in nearly 57 countries with India, Australia, Myanmar, Ethiopia, Turkey, and Russia as the major producers (Merga and Haji 2019). The crop production is severely affected by various biotic and abiotic stresses leading up to 90% yield losses (Kumar et al., 2018). Among biotic stresses, gram pod borer *Helicoverpa armigera* (Hübner) [Lepidoptera: Noctuidae] causes significant crop damage annually (90%) estimated at US $330 million worldwide (Rao et al., 2013; Patil et al., 2017). *H*. *armigera* is difficult to control as it has migratory behavior, numerous generations per year, adaptability to different environmental conditions, high fecundity, and insecticidal resistance (Fitt 1989). Furthermore, the biopesticides used to control the insect have high production costs coupled with poor product quality control systems (Cherry et al., 2000; Jenkins and Grzywacz 2000). The development of pod borer-resistant chickpea cultivars through conventional breeding is hampered due to the narrow crop genetic base and crossability barriers between cultivated chickpea and wild *Cicer* species (Mallikarjuna et al., 2007).

The pod borer larvae have been effectively controlled through specific insecticidal crystal proteins of *Bacillus thuringiensis,* and Cry1Ac is the most effective toxin against *H*. *armigera* (Chakrabarti et al., 1998). Cry1Ac protein acts by targeting the insect midgut in which the prevalence of high pH solubilizes the protein; the activated protein forms a pore complex in the insect epithelial membrane causing lysis and eventually larval death (Bravo et al., 2008). The pod borer attack has been countered efficiently by transgenic chickpea plants carrying cry1Ac, cry1Ab, cry2Aa, and cry1Aa3 (Kar et al., 1997; Sanyal et al., 2005; Acharjee et al., 2010; Mehrotra et al., 2011; Khatodia et al., 2014). The introgression of cry genes from transgenic plants into elite cultivars/lines through marker-assisted backcross breeding leads to precise trait transfer, for e.g., enhanced resistance against striped stem borer in rice by introgression of cry1Ab (Wang et al., 2012), improved resistance against corn borer with cry1A.105 and cry2ab2 in maize inbred lines (Venkatesh et al., 2015), increased tolerance to fruit/shoot borer in eggplant following cry1Ac transfer (Ripalda et al., 2012), and improved insect resistance in cotton *via* cryIA introgression (Guo et al., 2005), etc. Marker-assisted backcross breeding, an effective molecular breeding technique, enables the transfer of desirable genes from an agronomically inferior donor into an elite recipient in a few generations, without linkage drag and in a smaller population size (Hospital and Charcosset 1997).

The introgression of cry genes from transgenic chickpea lines to commercial chickpea cultivars for imparting resistance against *Helicoverpa* following marker-assisted backcross breeding is not reported so far. In the present study, an attempt was made to convert two elite but pod borer-susceptible chickpea cultivars, namely, PBG7 and L552, into resistant cultivars by introgressing cry1Ac from pod borer-resistant transgenic lines, namely, BS 100B and BS 100E through marker-assisted backcross breeding. PBG7 is a high-yielding cultivar of desi chickpea, whereas L552 is a bold-seeded high-yielding cultivar of kabuli chickpea; both cultivars are recommended for commercial cultivation in the North Indian state, Punjab, and possess good cooking quality (Sandhu et al., 2012; Singh et al., 2015). The backcross populations were analyzed for the presence of transgene, evaluated for Cry1Ac concentration, and bioassayed for toxicity to *H*. *armigera*. The highlighting feature of this study was the introgression of cry1Ac in BC_1_F_1_ populations and its subsequent transmission to BC_1_F_2_, BC_1_F_3_, BC_2_F_2_, and BC_2_F_3_ that displayed up to 100% *H*. *armigera* larval mortality, and agronomic performance of selected BC_2_F_2_ and BC_2_F_3_ plants was similar to that of the recurrent parent.

## Materials and Methods

### Plant Material

**FIGURE 1:**
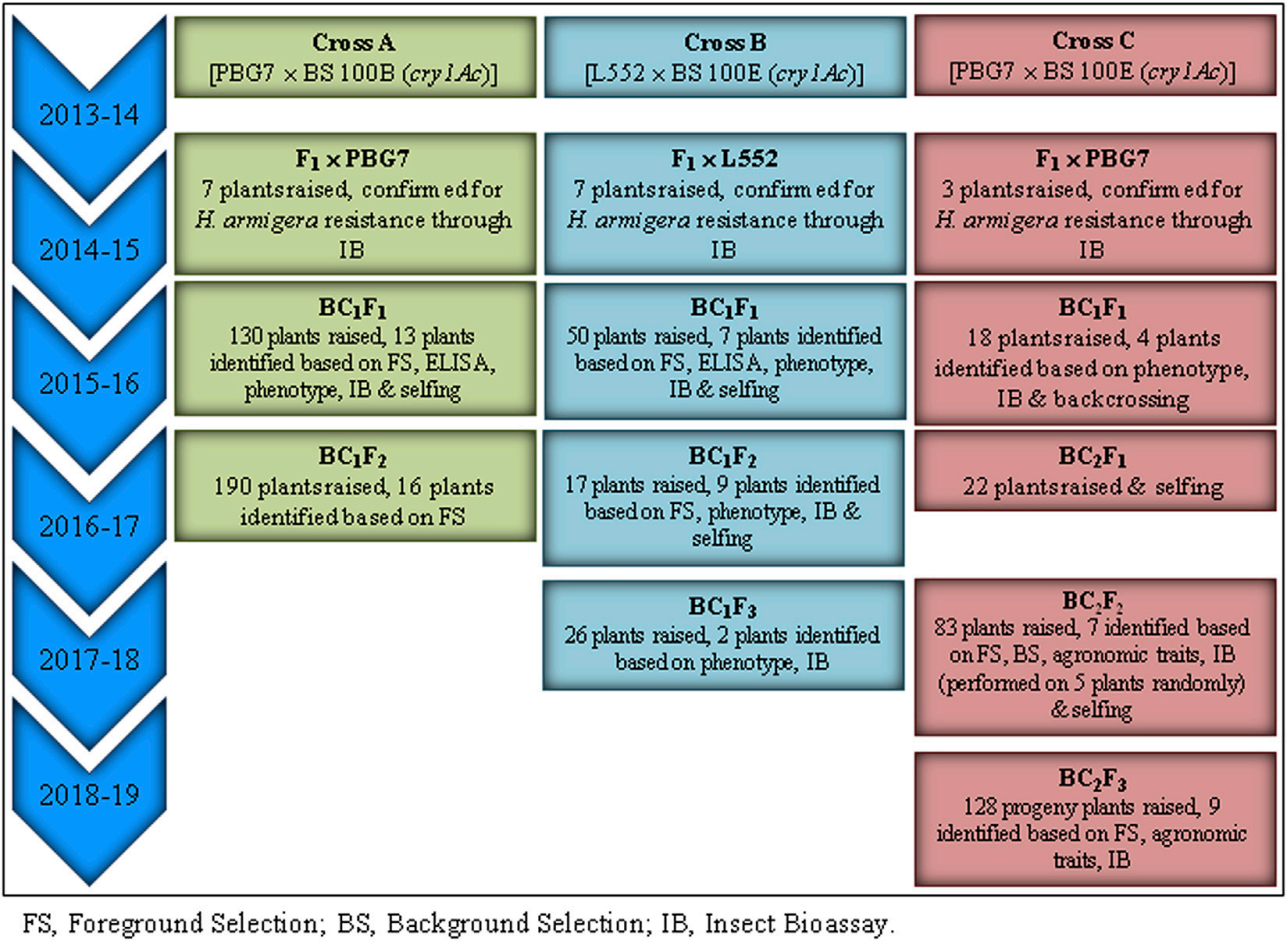
Schematic overview of marker-assisted backcross breeding of commercial chickpea cultivars × transgenic lines.

T_5_ seeds (15 in number) for each of two transgenic chickpea lines, namely, BS 100B and BS 100E expressing cry1Ac gene under the control of the *Arabidopsis* Rubisco small subunit gene promoter and tobacco SSU terminator (Supplementary Figure S1), were procured from the Department of Biotechnology-Assam Agricultural University Centre, Assam Agricultural University, Jorhat, Assam, India, during 2013. The transgenic lines carrying cry1Ac at a single locus were used as donor (male) parents in chickpea backcrossing program; the lines are reported to accumulate a high level of Cry1Ac protein (˃ 50 μg g^−1^ leaf tissue) that causes 80–100% neonatal *H*. *armigera* larval mortality (Hazarika et al., 2019). The high-yielding commercial cultivars PBG7 (desi) and L552 (kabuli) were used as recipient (female) parents. F_1_ plants of PBG7 × BS 100B (designated as Cross A), L552 × BS 100E (Cross B), and PBG7 × BS 100E (Cross C) were backcrossed with their respective recipient parents to obtain BC_1_F_1_ seeds that were sown to generate BC_1_F_1_ populations. F_1_ plants, BC_1_F_1_, BC_1_F_2_, BC_1_F_3_, BC_2_F_1_, BC_2_F_2,_ and BC_2_F_3_ populations were raised under contained conditions (Supplementary Figure S2; Supplementary Table S1) in a net house (30-mesh screen) at Experimental Farms, Department of Plant Breeding & Genetics, Punjab Agricultural University (PAU), Ludhiana. The populations were grown in plots comprising 25 rows with 2 m length and row-to-row distance of 40 cm following normal agronomic practices during the month of October. The schematic overview of marker assisted-backcross breeding of commercial chickpea cultivars with transgenic lines is shown in Figure 1.

### DNA Extraction

Genomic DNA was extracted from tender twigs of 20-day-old BC_1_F_1_, BC_1_F_2_, BC_2_F_2_, and BC_2_F_3_ populations, transgenic donor parents BS 100B and BS 100E, and non-transgenic recipient parents PBG7 and L552 according to the miniprep method. For quantification, the extracted DNA was electrophoresed on 0.8% (w/v) agarose gel using PowerPacHC (Bio-Rad, United States) at 50 V for 2 h; ethidium bromide-stained gel was visualized under UV light and photographed on a 110 V AlphaImager HP imaging system (ProteinSimple, United Kingdom).

### Foreground Selection for cry1Ac-Positive Plants

The foreground selection of backcross populations was carried out through PCR using cry1Ac-specific (Accession Number M11068, Hazarika et al., 2019), internal forward 5-TAT​CTT​TGG​TCC​ATC​TCA​ATG​GG-3 and reverse 5-GTG​TCC​AGA​CCA​GTA​ATA​CTC-3 primers to amplify 757 bp transgene. PCR mixture (20 µl) contained 50 ng genomic DNA (2 µl), 10 µM of each primer (0.6 µl), 1 mM dNTPs (4 µl), 25 mM MgCl_2_ (1.5 µl), 5 × Green GoTaq Flexi buffer (4 µl), 5 units GoTaq DNA polymerase (1 µl) [Promega, United States] and nuclease-free water (6.3 µl). The reaction mixtures were placed in a GeneAmp PCR System 9700 (Thermo Fisher Scientific, United States) programmed for an initial denaturation at 94 C for 4 min, followed by 35 cycles of denaturation at 94 C for 50 s, annealing at 58 C for 1 min, extension at 72 C for 1 min, and concluded by a final extension at 72 C for 7 min and held at 4 C prior to storage. The amplicons were resolved on 1.5% (w/v) agarose gel, visualized, and photographed. The statistical significance for cry1Ac segregation data was determined by Chi-square analysis using the formula: χ^2^ = (O-E)^2^/E, where O is the observed value and E is the expected value.

### Enzyme-Linked Immunosorbent Assay

Cry1Ac expression in BC_1_F_1_ plants and transgenic donor and non-transgenic recipient parents was quantified through ELISA using Cry1Ac QuantiPlate kit (EnviroLogix, United States) according to the manufacturer’s instructions. A total of two leaflets (10 mg) of each plant were homogenized in an Eppendorf grinding tube for 20–30 s by adding 500 µl of 1 × extraction buffer. Each leaf tissue sample (50 µl) was diluted in a 1:11 ratio by adding 550 µl of 1 × extraction buffer; thereafter, 100 µl each of diluted sample, negative control, and positive calibrator was dispensed in the ELISA plate, followed by parafilm masking and incubation at an ambient temperature for 15 min. The assay was performed in triplicate. Cry1Ac-enzyme conjugate (100 µl) was added to each well, and the plate was again covered with parafilm and incubated for 1 h. After incubation, the parafilm mask was removed and well contents were agitated vigorously to decant the wells. The vacant wells were flooded with washing buffer and agitated to decant; the washing step was performed thrice. Then substrate (100 µl) was added to each well and mixed thoroughly, followed by plate covering with parafilm and incubation for 20 min. The reaction was terminated by adding 100 µl of stop solution to each well. The ELISA plate was read in a 96-well ELISA plate reader Infinite 200 Pro (Tecan, Switzerland) at 450 and 600 nm. The optical density (OD) values of samples and positive calibrators were analyzed using a Microsoft Excel sheet to generate a linear scale graph of the mean OD of each calibrator against its Cry1Ac concentration (Supplementary Table S2). The amount of Cry1Ac protein in each leaf tissue sample (µg g^−1^) was determined using the formula {(OD of sample - mean OD of negative control) - 0.425/0.127} × dilution factor 1 (38.46) × dilution factor 2 (11)/1,000 (Supplementary Table S2). The data were analyzed for mean ± standard deviation using Microsoft Excel 2007 software at default settings.

### Background Selection for Recurrent Parent Genome Recovery

The background selection of cry1Ac-positive BC_2_F_2_ plants was carried out using Simple Sequence Repeat (SSR) markers. As a preliminary step, polymorphism analysis was undertaken on parents PBG7 and BS 100E using 210 markers belonging to the following series: CGMM, CaM, GA, GAA, TA, TAA, TS, TR, NCPGR, H, and CaSTMS 11 (Supplementary Table S3). The amplified products were resolved on 6% (w/v) PAGE, and marker data were scored based on differential separation of amplicon(s). BC_2_F_2_ plants possessing maximum recurrent parent genome were identified with reproducible polymorphic SSR markers (Supplementary Table S3). The percent recurrent parent genome recovery in a BC_2_F_2_ plant was calculated as the sum of the number of alleles corresponding to recurrent parent detected by polymorphic markers divided by the total number of alleles detected by polymorphic and cry1Ac-specific markers.

### Assessment for Agronomic Traits

The agronomic performance of BC_2_F_2_ population was assessed for plants analyzed for recurrent parent genome recovery, and of BC_2_F_3_ population was based on three progeny plants (from each BC_2_F_2_ plant) having phenotype similar to the recurrent parent. The data were recorded on days to 50% flowering, number of branches per plant, days to maturity, plant height, number of pods per plant, number of seeds per plant, 100-seed weight, biological yield, seed yield per plant, and harvest index, compared with the recurrent parent and analyzed for percent phenome recovery in BC_2_F_2_ plants and for mean ± standard deviation in BC_2_F_3_ plants.

### Bioassay for Determining Toxicity to *H*. *armigera*

Four-month-old morphologically healthy plants were analyzed for toxicity to *H*. *armigera* using two approaches, i.e., detached leaf bioassay and whole plant bioassay given by Sharma et al. (2005a), Sharma et al. (2005b) with modifications. Detached leaf bioassay: The terminal twigs having fully expanded leaflets were plucked from F_1_, backcross population (BC_1_F_1_, BC_1_F_2_ and BC_2_F_2_), transgenic donor parent and non-transgenic recipient parent plants, and placed on 3% (w/v) agar (HiMedia, India) medium slants in sterile 500 ml plastic cups and used for bioassay. *H*. *armigera* larvae (3rd to 4th instar) collected in February from chickpea fields were reared individually in bioassay cups and maintained initially on non-transformed tender chickpea twigs, followed by growth on a semi-synthetic diet (Armes et al., 1992) until pupation. The pupae were kept on moist sponges covered with filter paper (Whatman, United States) in plastic containers till the emergence of adults that were paired in oviposition chambers i.e., cell pots wrapped in black paper on all sides and covered with muslin cloth on top. The adults were fed on 5% (v/v) honey solution by hanging honey-soaked cotton swab inside each oviposition chamber. Subsequently, egg laying occurred on the muslin cloth that was shifted to bioassay cup containing semi-synthetic diet for egg hatching, thereafter, neonates were used for bioassay of plant twigs. Ten neonate larvae were released in each bioassay cup and incubated in a growth chamber (Saveer Biotech Limited, India) maintained at 25 ± 2 C, 14 h light: 10 h dark period and >65 ± 5 percent relative humidity. The bioassay was replicated thrice and performed in the Pulses Entomology Laboratory, Department of Plant Breeding & Genetics, PAU, Ludhiana. The assayed plants were visually scored for the damage caused by neonate larvae after 96 h of release on a scale of 1–9 (1 = < 10% leaf area damaged, and 9 = > 80% leaf area damaged) given by Sharma et al. (2005a) for detached leaf assay. The larval mortality rate was compared among plants of backcross populations and donor and recipient parents to monitor the relationship between percent larval mortality and Cry1Ac protein concentration. The data were analyzed for mean ± standard deviation.

Whole plant bioassay: The assay was carried out under net house conditions on plants grown in plots with row to row distance of 40 cm and plant to plant spacing of 10 cm, according to the method given by Sharma et al. (2005b) for screening chickpea against *H*. *armigera* under greenhouse conditions with modifications. The healthy plants at the flowering stage from BC_1_F_3_ and BC_2_F_3_ populations and donor and recipient parents were covered with cages sized 25 × 25 × 75 cm^3^. The cages made of galvanized iron wire (2 mm in diameter) were supported by four vertical bars and covered with a muslin cloth bag. The experiment was performed in triplicate by caging three plants of each population, parent individually and releasing 10 *H*. *armigera* neonatal larvae on each plant, and terminated after 120 h when significant leaf area was damaged in recipient parents. The plants were scored for leaf-feeding visually on a 1-9 scale (where 1 = ˂ 10%, 2 = 11–20%, 3 = 21–30%, 4 = 31–40%, 5 = 41–50%, 6 = 51–60%, 7 = 61–70%, 8 = 71–80% and 9 = > 80% leaf area and/or pods damaged). The number of surviving larvae was recorded and individually placed in 25 ml plastic cups to express the data as percent larval mortality that were analyzed using Microsoft Excel 2007 software. No insecticide was applied in the experiment.

### Statistical Analysis

Data on Cry1Ac protein concentration, leaf-feeding, and larval mortality in backcross populations are presented as mean ± SD of three replicates. Statistical significance for the segregation data was determined using Chi-square analysis; calculated Chi-square value > table value was considered statistically significant at 5 percent level of significance.

## Results

### Analysis on F_1_ Plants for Determining Toxicity to *H*. *armigera*

F_1_ plants developed from Cross A, Cross B, Cross C, and transgenic donor parent and non-transgenic recipient parents were analyzed for toxicity to *H*. *armigera*. F_1_ plants obtained from Cross A (seven in number), Cross B (seven), Cross C (three), and donor parent displayed 100% *H*. *armigera* neonatal larval mortality and negligible (<10–20%) leaf-feeding damage, whereas recipient parents exhibited 23.33–30% larval mortality on an average with significant (51 to 70%) leaf-feeding damage (Table 1). F_1_ plants toxic to *H*. *armigera* were backcrossed to generate BC_1_F_1_ populations.

**TABLE 1:** Bioassay on F_1_ plants and backcross populations raised by crossing commercial chickpea cultivars with cry1Ac transgenic lines for toxicity to *H*. *armigera* neonatal larvae.

S. No	Plant number/ parent	Cry1Ac protein concentration (µg G-1 leaf tissue)			Average protein concentration (µg G-1 leaf tissue)	Leaf feeding score^a^			Average leaf feeding score	Leaf feeding damage (%)	Larval mortality (%)			Average larval mortality (%)
		R_1_	R_2_	R_3_		R_1_	R_2_	R_3_			R_1_	R_2_	R_3_	
F_1_ plants (derived from Cross A) through detached leaf bioassay													
1	1	NA	NA	NA	NA	1	1	1	1 ± 0.0	˂ 10	100	100	100	100 ± 0.0
2	2	NA	NA	NA	NA	1	1	2	1.33 ± 0.58	˂ 10–20	100	100	100	100 ± 0.0
3	3	NA	NA	NA	NA	1	1	2	1.33 ± 0.58	˂ 10–20	100	100	100	100 ± 0.0
4	4	NA	NA	NA	NA	1	2	1	1.33 ± 0.58	˂ 10–20	100	100	100	100 ± 0.0
5	5	NA	NA	NA	NA	1	1	1	1 ± 0.0	˂ 10	100	100	100	100 ± 0.0
6	6	NA	NA	NA	NA	1	1	1	1 ± 0.0	˂ 10	100	100	100	100 ± 0.0
7	7	NA	NA	NA	NA	1	1	1	1 ± 0.0	˂ 10	100	100	100	100 ± 0.0
	BS 100B	NA	NA	NA	NA	1	1	1	1 ± 0.0	˂ 10	100	100	100	100 ± 0.0
	PBG7	NA	NA	NA	NA	6	6	6	6 ± 0.0	51–60	30	30	30	30 ± 0.0
F1 plants (derived from Cross B) through detached leaf bioassay													
1	1	NA	NA	NA	NA	2	1	1	1.33 ± 0.58	˂ 10–20	100	100	100	100 ± 0.0
2	2	NA	NA	NA	NA	2	1	1	1.33 ± 0.58	˂ 10–20	100	100	100	100 ± 0.0
3	3	NA	NA	NA	NA	1	2	1	1.33 ± 0.58	˂ 10–20	100	100	100	100 ± 0.0
4	4	NA	NA	NA	NA	1	1	2	1.33 ± 0.58	˂ 10–20	100	100	100	100 ± 0.0
5	5	NA	NA	NA	NA	1	1	1	1 ± 0.0	˂ 10	100	100	100	100 ± 0.0
6	6	NA	NA	NA	NA	1	1	1	1 ± 0.0	˂ 10	100	100	100	100 ± 0.0
7	7	NA	NA	NA	NA	1	2	2	1.67 ± 0.58	˂ 10–20	100	100	100	100 ± 0.0
	BS 100E	NA	NA	NA	NA	1	1	1	1 ± 0.0	˂ 10	100	100	100	100 ± 0.0
	L552	NA	NA	NA	NA	7	7	7	7 ± 0.0	61–70	30	20	20	23.33 ± 5.77
F1 plants (derived from Cross C) through detached leaf bioassay													
1	1	NA	NA	NA	NA	1	1	1	1 ± 0.0	˂ 10	100	100	100	100 ± 0.0
2	2	NA	NA	NA	NA	2	2	2	2 ± 0.0	11–20	100	100	100	100 ± 0.0
3	3	NA	NA	NA	NA	1	1	1	1 ± 0.0	˂ 10	100	100	100	100 ± 0.0
	BS 100E	NA	NA	NA	NA	1	1	1	1 ± 0.0	˂ 10	100	100	100	100 ± 0.0
	PBG7	NA	NA	NA	NA	6	6	6	6 ± 0.0	51–60	30	30	30	30 ± 0.0
BC_1_F_1_ population (derived from Cross A) through detached leaf bioassay													
1	4	11.68	11.71	11.71	11.70 ± 0.02	1	1	1	1 ± 0.0	˂ 10	100	100	100	100 ± 0.0
2	6	11.71	11.71	11.71	11.71 ± 0.0	1	1	1	1 ± 0.0	˂ 10	100	100	100	100 ± 0.0
3	7	11.27	11.28	11.27	11.27 ± 0.01	2	2	2	2 ± 0.0	11–20	100	100	100	100 ± 0.0
4	16	11.68	11.68	11.68	11.68 ± 0.0	2	2	2	2 ± 0.0	11–20	100	100	100	100 ± 0.0
5	17	11.71	11.71	11.71	11.71 ± 0.0	2	2	2	2 ± 0.0	11–20	100	100	100	100 ± 0.0
6	18	11.71	11.71	11.71	11.71 ± 0.0	2	2	2	2 ± 0.0	11–20	100	100	100	100 ± 0.0
7	21	11.57	11.61	11.61	11.60 ± 0.02	1	1	2	1.33 ± 0.58	˂ 10–20	100	100	100	100 ± 0.0
8	22	11.27	11.38	11.31	11.32 ± 0.06	2	2	2	2 ± 0.0	11–20	100	100	100	100 ± 0.0
9	24	11.64	11.61	11.64	11.63 ± 0.02	1	1	1	1 ± 0.0	˂ 10	100	100	100	100 ± 0.0
10	77	11.64	11.64	11.64	11.64 ± 0.0	1	1	1	1 ± 0.0	˂ 10	100	100	100	100 ± 0.0
11	81	11.34	11.34	11.34	11.34 ± 0.0	1	1	1	1 ± 0.0	˂ 10	100	100	100	100 ± 0.0
12	89	11.64	11.64	11.64	11.64 ± 0.0	1	1	1	1 ± 0.0	˂ 10	100	100	100	100 ± 0.0
13	90	11.04	11.01	11.04	11.03 ± 0.02	1	1	1	1 ± 0.0	˂ 10	100	100	100	100 ± 0.0
	BS 100B	11.38	11.34	11.34	11.35 ± 0.02	1	1	1	1 ± 0.0	˂ 10	100	100	100	100 ± 0.0
	PBG7	0	0	0	0 ± 0.0	6	6	6	6 ± 0.0	51–60	30	30	30	30 ± 0.0
BC_1_F_1_ population (derived from Cross B) through detached leaf bioassay													
1	1	11.61	11.61	11.61	11.61 ± 0.0	1	1	1	1 ± 0.0	˂ 10	100	100	100	100 ± 0.0
2	2	11.57	11.57	11.57	11.57 ± 0.0	1	1	1	1 ± 0.0	˂ 10	100	100	100	100 ± 0.0
3	4	11.51	11.51	11.51	11.51 ± 0.0	1	1	1	1 ± 0.0	˂ 10	100	100	100	100 ± 0.0
4	25	11.51	11.51	11.51	11.51 ± 0.0	1	2	1	1.33 ± 0.58	˂ 10–20	100	80	100	93.33 ± 11.55
5	34	11.68	11.68	11.68	11.68 ± 0.0	1	1	1	1 ± 0.0	˂ 10	100	100	100	100 ± 0.0
6	36	11.61	11.61	11.61	11.61 ± 0.0	1	2	1	1.33 ± 0.58	˂ 10–20	100	80	100	93.33 ± 11.55
7	41	11.68	11.68	11.68	11.68 ± 0.0	1	1	1	1 ± 0.0	˂ 10	100	100	100	100 ± 0.0
8	42	11.51	11.51	11.51	11.51 ± 0.0	1	1	1	1 ± 0.0	˂ 10	100	100	100	100 ± 0.0
9	45	11.54	11.51	11.54	11.53 ± 0.02	1	1	1	1 ± 0.0	˂ 10	100	100	100	100 ± 0.0
	BS 100E	11.68	11.61	11.64	11.64 ± 0.04	1	1	1	1 ± 0.0	˂ 10	100	100	100	100 ± 0.0
	L552	0	0	0	0 ± 0.0	7	7	7	7 ± 0.0	61–70	30	20	20	23.33 ± 5.77
BC1F1 population (derived from Cross C) through detached leaf bioassay													
1	1	NA	NA	NA	NA	1	1	1	1 ± 0.0	˂ 10	100	100	100	100 ± 0.0
2	2	NA	NA	NA	NA	1	2	1	1.33 ± 0.58	˂ 10–20	100	80	100	93.33 ± 11.55
3	3	NA	NA	NA	NA	1	1	1	1 ± 0.0	˂ 10	100	100	100	100 ± 0.0
4	4	NA	NA	NA	NA	1	1	1	1 ± 0.0	˂ 10	100	100	100	100 ± 0.0
5	8	NA	NA	NA	NA	1	2	1	1 ± 0.0	˂ 10	100	100	100	100 ± 0.0
	BS 100E	NA	NA	NA	NA	0	0	0	0 ± 0.0	0	100	100	100	100 ± 0.0
	PBG7	NA	NA	NA	NA	6	6	6	6 ± 0.0	51–60	30	30	30	30 ± 0.0
BC_1_F2 population (derived from Cross B) through detached leaf bioassay													
1	1	NA	NA	NA	NA	1	1	1	1 ± 0.0	˂ 10	100	100	100	100 ± 0.0
2	3	NA	NA	NA	NA	1	1	1	1 ± 0.0	˂ 10	100	100	100	100 ± 0.0
3	9	NA	NA	NA	NA	1	1	1	1 ± 0.0	˂ 10	100	100	100	100 ± 0.0
4	10	NA	NA	NA	NA	1	1	1	1 ± 0.0	˂ 10	100	100	100	100 ± 0.0
5	12	NA	NA	NA	NA	1	1	1	1 ± 0.0	˂ 10	100	100	100	100 ± 0.0
6	14	NA	NA	NA	NA	1	1	1	1 ± 0.0	˂ 10	100	100	100	100 ± 0.0
7	15	NA	NA	NA	NA	1	1	1	1 ± 0.0	˂ 10	100	100	100	100 ± 0.0
8	16	NA	NA	NA	NA	1	1	1	1 ± 0.0	˂ 10	100	100	100	100 ± 0.0
9	17	NA	NA	NA	NA	1	1	1	1 ± 0.0	˂ 10	100	100	100	100 ± 0.0
	BS 100E	NA	NA	NA	NA	0	0	0	0 ± 0.0	0	100	100	100	100 ± 0.0
	L552	NA	NA	NA	NA	7	7	7	7 ± 0.0	61–70	30	20	20	23.33 ± 5.77
BC_1_F_3_ population (derived from Cross B) through whole plant bioassay													
1	1	NA	NA	NA	NA	2	3	2	2.33 ± 0.58^€^	11–30^b^	70	40	60	56.67 ± 15.27
2	3	NA	NA	NA	NA	2	2	2	2 ± 0.0	11–20	60	80	60	66.67 ± 11.55
3	9	NA	NA	NA	NA	1	2	1	1.33 ± 0.58	˂ 10–20	100	80	100	93.33 ± 11.55
4	10	NA	NA	NA	NA	5	6	5	5.33 ± 0.58	41–60	20	10	20	16.67 ± 5.77
5	12	NA	NA	NA	NA	3	2	2	2.33 ± 0.58	11–30	60	80	80	73.33 ± 11.55
6	14	NA	NA	NA	NA	2	1	1	1.33 ± 0.58	˂ 10–20	80	100	100	93.33 ± 11.55
	BS 100E	NA	NA	NA	NA	1	1	1	1 ± 0.0	˂ 10	100	100	100	100 ± 0.0
	L552	NA	NA	NA	NA	7	7	7	7 ± 0.0	61–70	30	20	20	23.33 ± 5.77
BC_2_F_2_ population (derived from Cross C) through detached leaf bioassay													
1	2	NA	NA	NA	NA	1	1	1	1 ± 0.0	˂ 10	100	100	100	100 ± 0.0
2	8	NA	NA	NA	NA	1	1	1	1 ± 0.0	˂ 10	100	100	100	100 ± 0.0
3	20	NA	NA	NA	NA	1	1	1	1 ± 0.0	˂ 10	100	100	100	100 ± 0.0
4	33	NA	NA	NA	NA	1	1	1	1 ± 0.0	˂ 10	100	100	100	100 ± 0.0
5	39	NA	NA	NA	NA	1	1	1	1 ± 0.0	˂ 10	100	100	100	100 ± 0.0
	BS 100E	NA	NA	NA	NA	1	1	1	1 ± 0.0	˂ 10	100	100	100	100 ± 0.0
	PBG7	NA	NA	NA	NA	6	6	6	6 ± 0.0	51–60	0	0	0	0 ± 0.0
BC_2_F_3_ population (derived from Cross C) through whole plant bioassay													
1	2	NA	NA	NA	NA	3	4	3	3.33 ± 0.58^€^	21–40^b^	40	60	60	53.33 ± 11.55
2	8	NA	NA	NA	NA	3	4	3	3.33 ± 0.58	21–40	40	60	60	53.33 ± 11.55
3	20	NA	NA	NA	NA	2	3	2	2.33 ± 0.58	11–30	80	60	80	73.33 ± 11.55
4	26	NA	NA	NA	NA	1	1	1	1 ± 0.0	˂ 10	100	100	100	100 ± 0.0
5	33	NA	NA	NA	NA	3	2	2	2.33 ± 0.58	11–30	60	80	80	73.33 ± 11.55
6	39	NA	NA	NA	NA	1	1	1	1 ± 0.0	˂ 10	100	100	100	100 ± 0.0
7	44	NA	NA	NA	NA	1	1	1	1 ± 0.0	˂ 10	100	100	100	100 ± 0.0
	BS 100E	NA	NA	NA	NA	1	1	1	1 ± 0.0	˂ 10	100	100	100	100 ± 0.0
	PBG7	NA	NA	NA	NA	6	6	6	6 ± 0.0	51–60	0	0	0	0 ± 0.0

Data on Cry1Ac protein concentration, leaf feeding, and larval mortality are presented as mean ± SD of three replicates. Leaf feeding score and larval mortality in detached leaf bioassay were recorded after 96 h of incubation whereas in whole plant bioassay after 120 h.

a Leaf feeding score: The plants were scored visually for the extent of damage caused on a 1–9 scale, where 1 = ˂ 10% and 9 = ˃ 80% leaf area damaged in detached leaf bioassay (Sharma et al., 2005a), and in whole plant bioassay, 1 = ˂ 10%, 2 = 11–20%, 3 = 21–30%, 4 = 31–40%, 5 = 41–50%, 6 = 51–60%, 7 = 61–70%, 8 = 71–80%, and 9 = ˃ 80% leaf area and/or pods damaged (Sharma et al., 2005b). NA, not analyzed.

b Average leaf and/or pod feeding score.

c Percent leaf and/or pod feeding damage; Cross A: PBG7 × BS 100B; Cross B: L552 × BS 100E; Cross C: PBG7 × BS 100E.

### Analysis on BC_1_F_1_ Populations for Foreground Selection and Determining Toxicity to *H*. *armigera*

The foreground selection of two BC_1_F_1_ populations derived from Cross A and Cross B, comprising 130 and 50 plants, respectively, was carried out through PCR using cry1Ac-specific primers. An amplicon corresponding to cry1Ac was detected in 46 (35.38%) BC_1_F_1_ plants obtained from Cross A (Supplementary Figure S3; Supplementary Table S1). The transgene segregation in a ratio of 1:1.8 deviated significantly from the 1:1 ratio expected if transgene was inserted at a single locus (Table 2). BC_1_F_1_ raised from Cross B segregated for the transgene in an expected Mendelian ratio of 1:1, as 25 (50.0%) plants were found to be cry1Ac positive (Supplementary Figure S4).

**TABLE 2:** Segregation analyses of backcross populations developed by crossing commercial chickpea cultivars with cry1Ac transgenic lines.

S. No	Backcross population	Observed number		Observed ratio	Expected number		Expected Ratio	Calculated χ^2^ value	*p*-value
		cry1Ac-positive plants	cry1Ac-negative plants		cry1Ac-positive plants	cry1Ac-negative plants			
1	BC_1_F_1_ (derived from Cross A)	46	84	1:1.8	65	65	1:1	11.10^a^	0.000863
2	BC_1_F_1_ (derived from Cross B)	25	25	1:1	25	25	1:1	0.0	1
3	BC_1_F_2_ (derived from Cross A)	16	174	1:10.9	142.50	47.50	3:1	449.18^a^	0.00001
4	BC_1_F_2_ (derived from Cross B)	13	4	3.3:1	12.75	4.25	3:1	0.02	0.8875
5	BC_2_F_2_ (derived from Cross C)	10	73	1:7.3	62.25	20.75	3:1	175.43^a^	0.00001
6	BC_2_F_3_ (derived from Cross C) progeny of: BC_2_F_2_ plant no. 1	18	3	6:1	15.75	5.25	3:1	1.28	0.257,899
	BC_2_F_2_ plant no. 2	7	3	2.3:1	7.50	2.50	3:1	0.13	0.718
	BC_2_F_2_ plant no. 8	5	4	1.3:1	6.75	2.25	3:1	1.81	0.1785
	BC_2_F_2_ plant no. 9	12	4	3:1	12	4	3:1	0.0	1
	BC_2_F_2_ plant no. 12	8	5	1.6:1	9.75	3.25	3:1	1.25	0.26355
	BC_2_F_2_ plant no. 20	4	1	4:1	3.75	1.25	3:1	0.07	0.791
	BC_2_F_2_ plant no. 26	16	0	16:0^§^	-	-	-	-	-
	BC_2_F_2_ plant no. 33	6	2	3:1	6	2	3:1	0.0	1
	BC_2_F_2_ plant no. 39	10	0	10:0^§^	-	-	-	-	-
	BC_2_F_2_ plant no. 44	20	0	20:0^§^	-	-	-	-	-

Data expressed as the number of cry1Ac-positive and -negative plants based on PCR.

a indicates significance at 0.05 level (χ^2^ table value = 3.84, 1 df)

b implies homozygous nature of respective BC_2_F_2_ plants; Cross A: PBG7 × BS 100B; Cross B: L552 × BS 100E; Cross C: PBG7 × BS 100E.

The recombinant protein concentration was estimated in 13 healthy BC_1_F_1_ plants derived from Cross A showing amplification of cry1Ac, nine Cross B plants along with transgenic donor parents BS 100B and BS 100E, and non-transgenic recipient parents PBG7 and L552 through ELISA (Supplementary Table S2). The average Cry1Ac protein concentration in both populations (11.03 to 11.71 μg g^−1^ leaf tissue) was at par with donor parents (11.35 to 11.64 μg g^−1^), whereas recipient parents did not exhibit any Cry1Ac concentration (Table 1). The BC_1_F_1_ plants (13 obtained from Cross A, nine from Cross B, and five from Cross C) had a phenotype similar to the recurrent parent, their bioassay for toxicity to *H*. *armigera* revealed that 13, 7, 4 plants from respective crosses, donor parents showed 100% *H*. *armigera* mortality and minor (<10–20%) leaf-feeding damage; in contrast, recipient parents exhibited 23.33–30% larval mortality with significant (51 to 70%) leaf-feeding damage (Table 1; Supplementary Figure S5). BC_1_F_1_ plants displaying toxicity to *H*. *armigera* were advanced for raising BC_1_F_2_ populations.

### Analysis on BC_1_F_2_ Populations for Foreground Selection and Determining Toxicity to *H*. *armigera*

The foreground selection was carried out on two BC_1_F_2_ populations: the first comprising of 190 plants derived from Cross A, and the second consisting of 17 plants obtained from Cross B; cry1Ac amplification was detected in 16 (8.4%) and 13 (76.5%) plants (Supplementary Figures S6, S7; Supplementary Table S1), exhibiting non-Mendelian (1:10.9) and Mendelian (3.3:1) segregation ratios in the two populations, respectively (Table 2).

The insect bioassay was performed on nine BC_1_F_2_ plants raised from Cross B displaying phenotypic growth similar to the recurrent parent, along with transgenic donor and non-transgenic recipient parents. In the BC_1_F_2_ plants, donor parent displayed 100% *H*. *armigera* mortality and negligible (<10%) leaf-feeding damage; however, recipient parent showed 23.33% larval mortality with significant (61 to 70%) leaf-feeding damage (Table 1). BC_1_F_2_ plants showing toxicity to *H*. *armigera* were used to raise BC_1_F_3_ population.

### Analysis on BC_1_F_3_ Population for Determining Toxicity to *H*. *armigera*

Six out of 26 BC_1_F_3_ plants having comparable phenotype to the recurrent parent developed from Cross B, transgenic donor parent, and non-transgenic recipient parent were analyzed for toxicity to *H*. *armigera*. The plants revealed 16.67–93.33% larval mortality and variable (˂ 10–60%) leaf and pod feeding damage; donor parent exhibited 100% *H*. *armigera* mortality with negligible (<10%) leaf and pod feeding damage, whereas recipient parent showed 23.33% larval mortality and significant (61 to 70%) damage to leaves and pods (Table 1). Two BC_1_F_3_ plants were observed to display 93.33% insect mortality.

### Analysis on BC_2_F_2_ Population for Foreground and Background Selection and Determining Toxicity to *H*. *armigera*.

The foreground selection of BC_2_F_2_ population derived from Cross C and comprising of 83 plants led to the identification of 10 (12.05%) plants showing amplification of cry1Ac (Supplementary Table S1). The population deviated significantly for transgene segregation (1:7.3) from the Mendelian ratio (3:1) for a single insertion site (Table 2).

**FIGURE 2:**
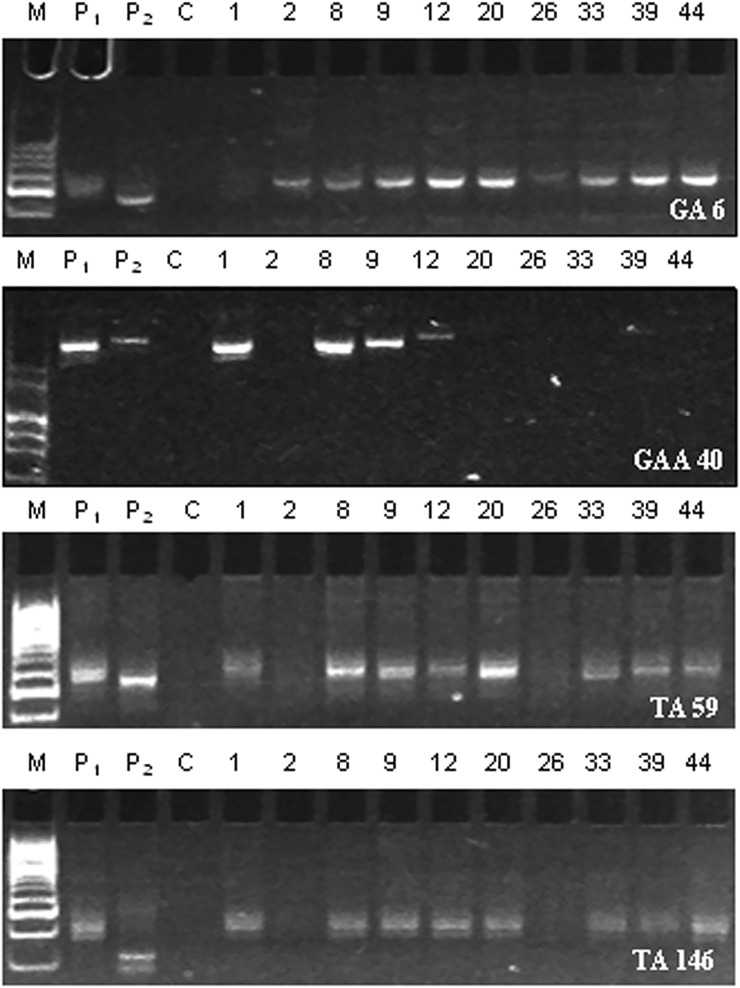
SSR amplification profiles of BC_2_F_2_ plants using polymorphic markers, namely, GA 6, GAA 40, TA 59, and TA 146. P_1_ indicates non-transgenic recipient parent PBG7; P_2_ represents transgenic donor parent BS 100E; C refers to control PCR reaction without template DNA; the numbers 1, 2, 8, 9, 12, 20, 26, 33, 39, and 44 denote BC_2_F_2_ plants; and M represents 50 bp DNA ladder (Cat. No. DM1100, SMOBIO Technology, Inc., Taiwan).

The donor and recipient parents were assessed for polymorphism using 210 SSR markers leading to the identification of 25 (11.9%) polymorphic markers (Supplementary Figure S8; Supplementary Table S3). The background selection using reproducible polymorphic markers on cry1Ac-positive BC_2_F_2_ plants demonstrated amplification pattern in ten BC_2_F_2_ plants (designated as 1, 2, 8, 9, 12, 20, 26, 33, 39, and 44) to be similar to recurrent parent “PBG7” profile (Figure 2), and the average recurrent parent genome recovery in these plants after two backcrosses was calculated to be 91.3% (Supplementary Table S4). The comparison of agronomic traits in BC_2_F_2_ plants with PBG7 revealed an average recurrent parent phenome recovery of 90.94% in BC_2_F_2_ plants (Table 3; Supplementary Table S5).

**TABLE 3:** Agronomic traits of BC_2_F_2_ and BC_2_F_3_ populations derived from Cross C (PBG7 × BS 100E).

S. No	Plant number/ parent	Agronomic trait										Recurrent parent phenome recovery (%)
		Days to 50% flowering	Number of branches per plant	Days to maturity	Plant height (cm)	Number of pods per plant	Number of seeds per plant	100-seed weight (g)	Biological yield (g)	Seed yield per plant (g)	Harvest index^a^ (%)	
BC_2_F_2_										
1	1	84 (93.33)	13 (76.47)	149 (96.13)	51.3 (86.51)	44 (73.33)	80 (70.80)	15.6 (93.97)	40.22 (77.97)	13.21 (76.36)	32.84 (97.91)	84.28
2	2	89 (98.89)	15 (88.23)	153 (98.71)	55.5 (93.59)	55 (91.67)	102 (90.26)	15.5 (93.37)	47.16 (91.43)	15.45 (89.31)	32.76 (97.67)	93.31
3	8	87 (96.67)	16 (94.12)	152 (98.06)	57.5 (96.96)	46 (76.67)	85 (75.22)	16.2 (97.59)	44.36 (86.00)	14.12 (81.62)	31.83 (94.90)	89.78
4	9	88 (97.78)	12 (70.59)	154 (99.35)	58.1 (97.98)	43 (71.67)	78 (69.03)	15.8 (95.18)	38.34 (74.33)	12.43 (71.85)	32.42 (96.66)	84.44
5	12	85 (94.44)	12 (70.59)	151 (97.42)	50.2 (84.65)	49 (81.67)	94 (83.18)	15.6 (93.97)	43.78 (84.88)	14.32 (82.77)	32.71 (97.52)	87.11
6	20	89 (98.89)	15 (88.23)	153 (98.71)	58.3 (98.31)	53 (88.33)	98 (86.72)	15.1 (90.96)	49.56 (96.08)	15.20 (87.86)	30.67 (91.44)	92.55
7	26	89 (98.89)	14 (82.35)	154 (99.35)	57.6 (97.13)	59 (98.33)	112 (99.11)	15.2 (91.57)	51.00 (98.87)	16.86 (97.46)	33.06 (97.46)	96.05
8	33	86 (95.55)	16 (94.12)	150 (96.77)	51.4 (86.68)	54 (90.00)	99 (87.61)	15.3 (92.17)	47.62 (92.32)	15.23 (88.03)	31.98 (95.35)	91.86
9	39	87 (96.67)	15 (88.23)	151 (97.42)	55.6 (93.76)	57 (95.00)	108 (95.57)	15.3 (92.17)	50.50 (97.91)	16.38 (94.68)	32.43 (96.69)	94.81
10	44	86 (95.55)	15 (88.23)	151 (97.42)	56.2 (94.77)	58 (96.67)	108 (95.57)	15.3 (92.17)	51.02 (98.91)	16.60 (95.95)	32.54 (97.02)	95.23
	BS 100E	82	8	145	48.4	16	28	13.3	23.30	4.13	17.72	-
	PBG7	90	17	155	59.3	60	113	16.6	51.58	17.30	33.54	-
Average recurrent parent phenome recovery = 90.94%										
BC_2_F_3_										
1	2	88.33 ± 0.58	14.00 ± 1.00	150.67 ± 0.58	55.70 ± 1.58	50.67 ± 2.52	97.67 ± 3.51	15.36 ± 0.21	44.34 ± 2.78	13.99 ± 1.12	31.59 ± 2.59	
2	8	86.00 ± 1.00	16.00 ± 1.00	151.67 ± 1.15	55.80 ± 2.66	44.33 ± 2.08	83.33 ± 5.51	15.83 ± 0.15	44.07 ± 3.09	14.28 ± 0.16	32.50 ± 2.04	
3	20	87.33 ± 1.15	15.67 ± 0.58	154.30 ± 0.58	57.47 ± 2.20	53.67 ± 1.53	99.00 ± 1.00	15.37 ± 0.49	52.08 ± 2.08	15.64 ± 0.51	30.03 ± 0.26	
4	26	89.00 ± 1.00	13.67 ± 0.58	152.00 ± 0.27	58.13 ± 2.37	54.00 ± 3.00	111.00 ± 4.58	15.13 ± 0.35	52.13 ± 3.56	15.33 ± 0.98	31.35 ± 0.27	
5	33	86.67 ± 1.53	14.00 ± 1.00	152.67 ± 1.15	55.30 ± 4.05	44.00 ± 3.00	79.33 ± 4.16	15.60 ± 0.26	39.42 ± 2.61	12.88 ± 0.22	32.77 ± 1.86	
6	39	86.67 ± 1.53	15.33 ± 1.15	152.33 ± 1.53	53.76 ± 3.16	55.00 ± 4.00	103.67 ± 7.64	15.23 ± 0.66	48.36 ± 1.61	15.74 ± 0.62	32.55 ± 0.66	
7	44	86.33 ± 1.53	14.00 ± 1.00	150.67 ± 0.58	57.03 ± 2.05	53.67 ± 3.78	102.67 ± 7.02	15.23 ± 0.55	45.20 ± 1.16	14.84 ± 0.53	32.83 ± 0.56	
	BS 100E	82.33 ± 1.15	8.33 ± 1.15	147.00 ± 1.00	46.50 ± 2.52	14.67 ± 2.08	25.33 ± 3.51	13.40 ± 0.43	22.71 ± 1.63	4.29 ± 0.22	18.94 ± 0.64	
	PBG7	88.33 ± 0.58	16.33 ± 0.58	154.00 ± 1.00	57.13 ± 2.43	54.67 ± 3.05	107.00 ± 7.00	15.93 ± 0.25	49.54 ± 1.91	15.90 ± 0.56	32.10 ± 0.22	

Data on BC_2_F_2_ population are presented for the plants analyzed for recurrent parent genome recovery; Data on BC_2_F_3_ population are based on three plants phenotypically similar to PBG7 and presented as mean ± SD; figures in parentheses are recurrent parent recovery percentages for agronomic traits calculated as plant trait value/value of PBG7 for that trait × 100.

a Harvest index = seed yield per plant/biological yield × 100; recurrent parent phenome recovery percentage was calculated as the sum of recurrent parent recovery percentages for different traits/10.

The randomly selected BC_2_F_2_ plants (designated as 2, 8, 20, 33, and 39) were bioassayed for toxicity to *H*. *armigera*. The results revealed that the selected plants and transgenic donor parent exhibited 100% larval mortality and negligible (<10%) leaf-feeding damage, whereas non-transgenic recipient parent was vulnerable to *H*. *armigera* with no larval mortality and significant (51 to 60%) leaf-feeding damage (Table 1). Subsequently, seeds of all ten BC_2_F_2_ plants were sown to obtain BC_2_F_3_ population.

### Analysis on BC_2_F_3_ Population for Foreground Selection, Agronomic Traits, and Determining Toxicity to *H*. *armigera*.

**FIGURE 3:**
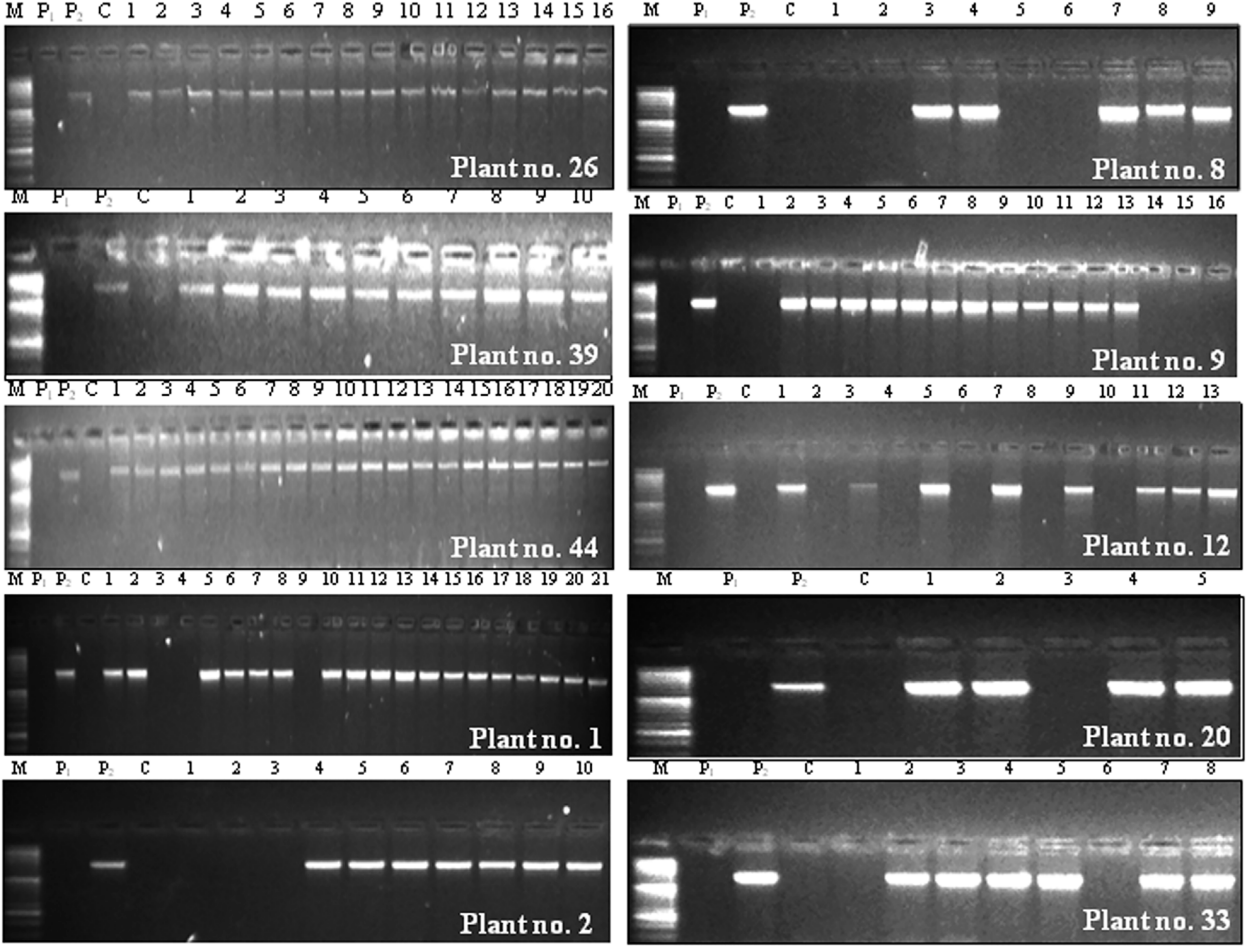
Foreground selection of BC_2_F_3_ population derived from Cross C (PBG7 × BS 100E) through PCR using cry1Ac-specific primers. P_1_ indicates non-transgenic recipient parent PBG7; P_2_ represents transgenic donor parent BS 100E; C refers to control PCR reaction without template DNA; the numbers on top of each gel represent BC_2_F_3_ progenies of a specific plant, and its identity is mentioned in the right bottom corner of each gel; the plants designated as 26, 39, and 44 were homozygous for cry1Ac, and those designated as 1, 2, 8, 9, 12, 20, and 33 were hemizygous for the transgene; M represents 50 bp DNA ladder (Cat. No. DM1100).

BC_2_F_3_ population obtained from Cross C, consisting of 128 plants was subjected to foreground selection for identifying BC_2_F_2_ plants homozygous for cry1Ac through recognition of BC_2_F_3_ plants carrying cry1Ac gene. The results revealed that on an average, 82.81% BC_2_F_3_ plants carried cry1Ac, and three (30%) BC_2_F_2_ plants designated as 26, 39, and 44 were homozygous for the transgene as all progeny plants (16 of plant no. 26, 10 of plant no. 39, and 20 of plant no. 44) contained the transgene (Table 2: Figure 3). On the contrary, the remaining seven BC_2_F_2_ plants designated as 1, 2, 8, 9, 12, 20, and 33 were hemizygous for cry1Ac with their BC_2_F_3_ progeny plants segregating in a ratio of 6:1, 2.3:1, 1.3:1, 3:1, 1.6:1, 4:1, and 3:1, respectively for transgenes that were found to fit in Mendelian 3:1 ratio expected for a selfed population (Table 2).

BC_2_F_3_ progeny plants belonging to seven BC_2_F_2_ plants, namely 2, 8, 20, 26, 33, 39, and 44, were assessed for agronomic performance. The results showed that the mean number of pods and seed yield of BC_2_F_3_ progeny plants derived from BC_2_F_2_ plant no. 20 and homozygous BC_2_F_2_ plants, namely 26, 39, and 44 were 53.67 ± 1.53, 54.00 ± 3.00, 55.00 ± 4.00, 53.67 ± 3.78, and 15.64 ± 0.51 g, 15.33 ± 0.98 g, 15.74 ± 0.62 g, 14.84 ± 0.53 g, respectively were statistically similar to mean number of pods (54.67 ± 3.05) and seed yield (15.90 ± 0.56 g) of recurrent parent (PBG 7) (Table 3; Supplementary Table S5).

**FIGURE 4:**
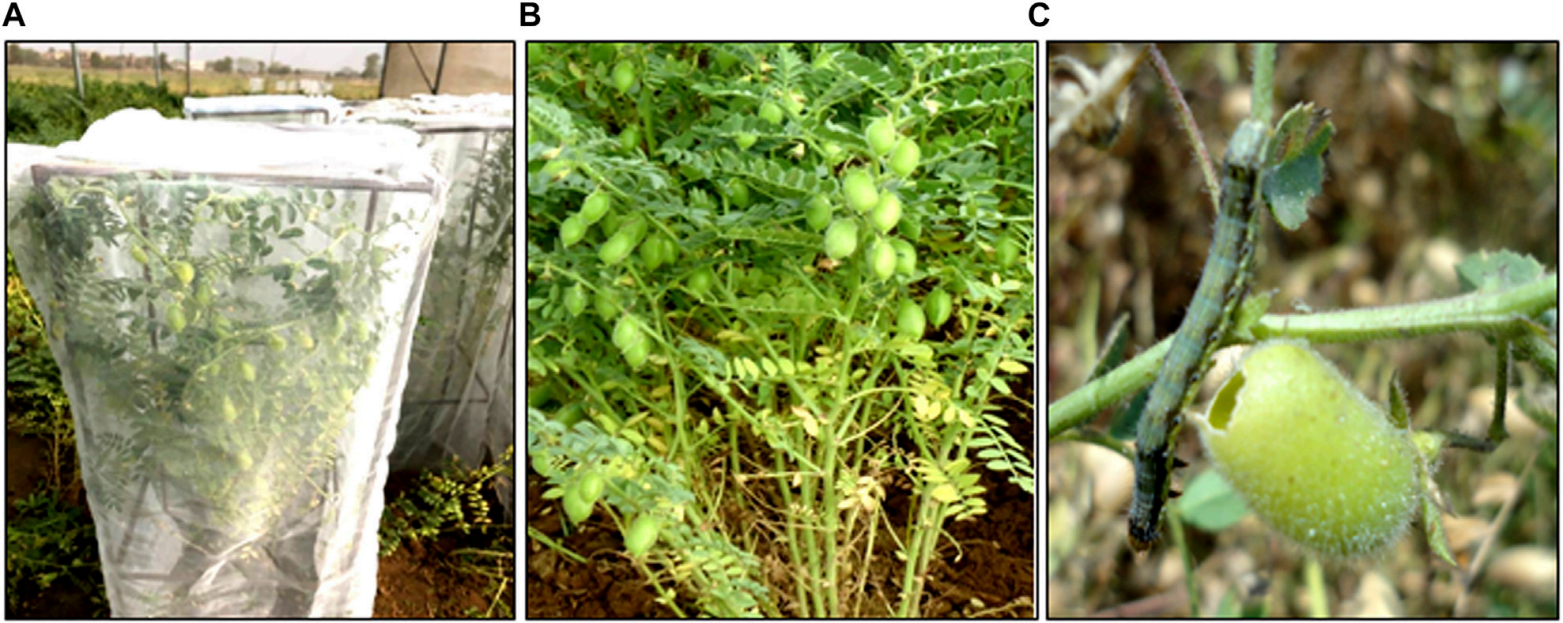
Bioassay on BC_2_F_3_ plants obtained from Cross C (PBG7 × BS 100E) expressing cry1Ac for toxicity to *H*. *armigera* through whole plant screening. **(A)** Caged plants displaying healthy leaves and pods. **(B)** Closer view of plant showing healthy leaves and pods. **(C)** Non-transgenic recipient parent PBG7 exhibiting damaged pod and surviving larva.

The bioassay of BC_2_F_3_ progeny plants revealed 53.33–100% *H*. *armigera* larval mortality and variable (<10–40%) leaf and pod feeding damage; amongst these, the progeny of homozygous BC_2_F_2_ plants displayed 100% mortality with negligible (<10%) leaf and pod feeding damage (Table 1; Figure 4A,B). The larval mortality in transgenic donor parent and BC_2_F_3_ progeny plants was similar, whereas the non-transgenic recipient parent displayed no larval mortality and significant (51 to 60%) leaf, pod feeding damage (Figure 4C).

## Discussion

The elite, commercial chickpea cultivars susceptible to pod borer were converted into resistant by introgressing cry1Ac from transgenic lines through marker-assisted backcross breeding. F_1_ plants and their backcross populations i.e., BC_1_F_1,_ BC_1_F_2_, BC_1_F_3_, BC_2_F_2,_ and BC_2_F_3,_ exhibited up to 100% *H*. *armigera* neonatal larval mortality with agronomic performance similar to that of the recurrent parent. The high larval mortality was a result of Cry1Ac protein accumulation up to 11.00 μg g^−1^ in backcross populations; Bt protein concentration as low as 0.9–3.1 μg g^−1^ is reported to be highly insecticidal to corn earworm, *Helicoverpa* spp. in backcross populations of *Brassica napus* lines × wild *B*. *rapa* (Halfhill et al., 2001; Zhu et al., 2004). Zhang et al. (2012) and Chen et al. (2018) demonstrated that an even lower Cry1Ac concentration (0.5 to 1.2 μg g^−1^) in the artificial diet of *H*. *armigera* larvae induced distinct histopathological changes in goblet cells of larval midgut epithelial lining, such as breakage of microvilli, endoplasmic reticulum, disorganization of mitochondria and chromatin, 2–36 h after Cry1Ac ingestion that eventually caused mortality.

BC_1_F_1_ and BC_1_F_2_ populations derived from Cross B segregated in Mendelian ratios of 1:1 and 3:1, respectively, for cry1Ac under contained field conditions; similarly, BC_2_F_3_ progenies of hemizygous BC_2_F_2_ plants raised from Cross C also segregated in Mendelian ratio of 3:1, pointing toward stable inheritance of cry1Ac as a single dominant gene in plants of different backcross populations. The typical 3:1 segregation ratio in selfed population and 1:1 in backcross population (Peng et al., 1992; Datta et al., 1998) often results from the insertion of one copy of the foreign gene in the host genome. The introgression of cry1Ab transgene following marker-assisted breeding has been reported in BC_2_F_2_ and BC_1_ generations of cotton and rice, respectively (Agbios-Agriculture & Biotechnology Strategies (Canada), Inc., 2007; Kiani et al., 2009). The recurrent parent genome recovery in BC_2_F_2_ plants was higher (91.3%) in this study as compared to 87.5% genetic similarity to the recurrent parent obtained after two backcrosses through conventional breeding (Venkatesh et al., 2015). A recurrent parent genome recovery of 95.9% in BC_2_F_2_ rice plants was reported using polymorphic SSR markers by Chukwu et al. (2020). The marker-assisted backcross breeding is a dynamic approach for conveniently recognizing plants that have recovered over 98% of the recurrent parent genome in two to three backcross generations depending upon the availability of polymorphic markers (Stojsin 2010). The similarity of BC_2_F_2_ plants for agronomic traits with the recurrent parent in our study pointed toward the recurrent parent genome recovery, suggesting that in a situation where a limited number of polymorphic markers is available, the phenotypic characterization for agronomic traits is important. Joseph et al. (2004) reported that phenotypic selection coupled with fewer polymorphic markers between the parental lines maximizes recurrent parent genome recovery. We observed that the agronomic performance of BC_2_F_3_ progeny plants (derived from homozygous BC_2_F_2_ plants 26, 39 and 44) for main traits i.e., the number of pods and seed yield was statistically similar to the recurrent parent. Likewise, marker-assisted breeding between β-carotene-rich inbred lines UMI1200β^+^, UMI1230β^+^ × HKI163 in maize resulted in the development of improved BC_2_F_3_ lines exhibiting agronomic traits e.g., cob weight and single plant yield similar to the recurrent parents (Chandran et al., 2019).

The distorted segregation ratios were detected in BC_1_F_1_ and BC_1_F_2_ populations developed from Cross A. The distorted ratios generally arise due to transgene inactivation (Matzke and Matzke 1995), low viability/fertilization ability of transgenic pollen (Zhang et al., 1996), reduced germination (Sachs et al., 1998), genetic background (Scott et al., 1998; Wu et al., 2002), recessive lethal (Scott et al., 1998) etc. In the present study, segregation distortion in BC_1_F_1_ and BC_1_F_2_ populations might be a result of reduced germination and not due to 1) transgene inactivation: as cry1Ac amplicon was observed in 46 plants from a total of 130 BC_1_F_1_ plants, and 16 out of 190 BC_1_F_2_ plants 2) low viability/fertility of transgenic pollen: as the pollen from recipient parent PBG7 (and not from the transgenic line) was used to pollinate F_1_ plants to obtain BC_1_F_1_ plants; and further both BC_1_F_1_ and BC_1_F_2_ populations had resulted from a cross between desi PBG7 and desi BS 100B. Wu et al. (2002) observed that crosses between *japonica* and *japonica* rice had no significant effect on segregation ratios of cry1Ab, whereas *japonica* × *indica* resulted in distorted gene segregation in F_2_ population, 3) genetic background: as both PBG7 and BS 100B are desi chickpeas, or 4) recessive lethal: as BC_1_F_1_ plants were hemizygous in nature for cry1Ac. Our assumption of reduced germination responsible for distorted segregation ratios draws support from observations by Sachs et al. (1998) on non-Mendelian segregation of cry1Ac in F_2_ populations derived from MON 249 × CAMD-E due to failure of a large number of F_2_ seeds inheriting cry1Ac to germinate. They further suggested that reduced germination associated with the inheritance of cry1Ac in MON 249 plants was a result of direct insertion effect leading to silencing of one or more native genes. In our case, we conclude that reduced germination was possibly associated with the inheritance of cry1Ac gene present in BS 100B plants. cry1Ac integration in the genomes of homozygous BC_2_F_2_ plants and BC_2_F_3_ progeny plants is probably at the same position as backcross populations obtained from a single transformation event are reported to carry transgene at a constant position in the genomes (Bakó et al., 2013).

The genetic background of BC_1_F_1_ populations raised from Cross A and Cross B did not affect the transgene expression as Cry1Ac protein concentration in the two BC_1_F_1_ populations was similar to each other and transgenic donor line. This observation is consistent with reports on hybrids of *Bt* maize (Fearing et al., 1997; Bakó et al., 2013) and eggplant (Ripalda et al., 2012) producing a similar amount of Cry protein in backcross populations irrespective of genetic background. However, this might not always be true as Sachs et al. (1998) observed that cryIA gene expression in cotton lines was influenced by the background genotype. The backcross populations of chickpea F_1_ plants displayed a high degree of resistance to pod borer as compared to PBG7 and L552, implying stable expression of Cry1Ac throughout different generations.

In conclusion, cry1Ac was introgressed from transgenic chickpea lines into commercial cultivars through marker-assisted backcross breeding for imparting pod borer resistance; consequently, the backcross populations exhibited up to 100% *H*. *armigera* larval mortality. The BC_2_F_2_ plants homozygous for cry1Ac with high recurrent parent phenome recovery were identified; their BC_2_F_3_ progeny plants displaying agronomic performance similar to the recurrent parent are a valuable source of *H*. *armigera* resistance and can be used in chickpea breeding programs.

## Data Availability

The original contributions presented in the study are included in the article/Supplementary Material, further inquiries can be directed to the corresponding author.
