# Supplementary material for: Introgressing cry1Ac for Pod Borer Resistance in Chickpea Through Marker-Assisted Backcross Breeding
Source: Front Genet. 2022 Apr 12;13:847647. doi: 10.3389/fgene.2022.847647 (PMC9039336; doi:10.3389/fgene.2022.847647)
Supplement: Supplementary file 1 [file DataSheet1.PDF]

Introgressing *cry1Ac* for Pod Borer Resistance in Chickpea through Marker Assisted Backcross Breeding

***Ajinder Kaur*[1], *Urvashi Sharma*[1], *Sarvjeet Singh*[2], *Ravinder Singh*[2], *Yogesh Vikal*[1], *Satnam Singh*[3], *Palvi Malik*[1], *Khushpreet Kaur*[3], *Inderjit Singh*[2], *Shayla Bindra*[2], *Bidyut Kumar Sarmah*[4] and *Jagdeep Singh Sandhu*[1*]**

[1] *School of Agricultural Biotechnology, Punjab Agricultural University, Ludhiana, Punjab 141004, India,*
[2] *Pulses Section, Department of Plant Breeding and Genetics, Punjab Agricultural University, Ludhiana, Punjab 141004, India,* [3]*Punjab Agricultural University, Regional Research Station, Faridkot, Punjab 151203, India,* [4] *Department of Biotechnology-Assam Agricultural University Centre, Assam Agricultural University, Jorhat, Assam 785013, India*

[*] **Correspondence:** *Jagdeep Singh Sandhu*

js_sandhu@pau.edu

SUPPLEMENTARY FIGURE 1 Schematic map of transgene construct showing *cry1Ac* under the control of *Arabidopsis* SSU promoter and tobacco SSU terminator. RB is right border, LB is left border.

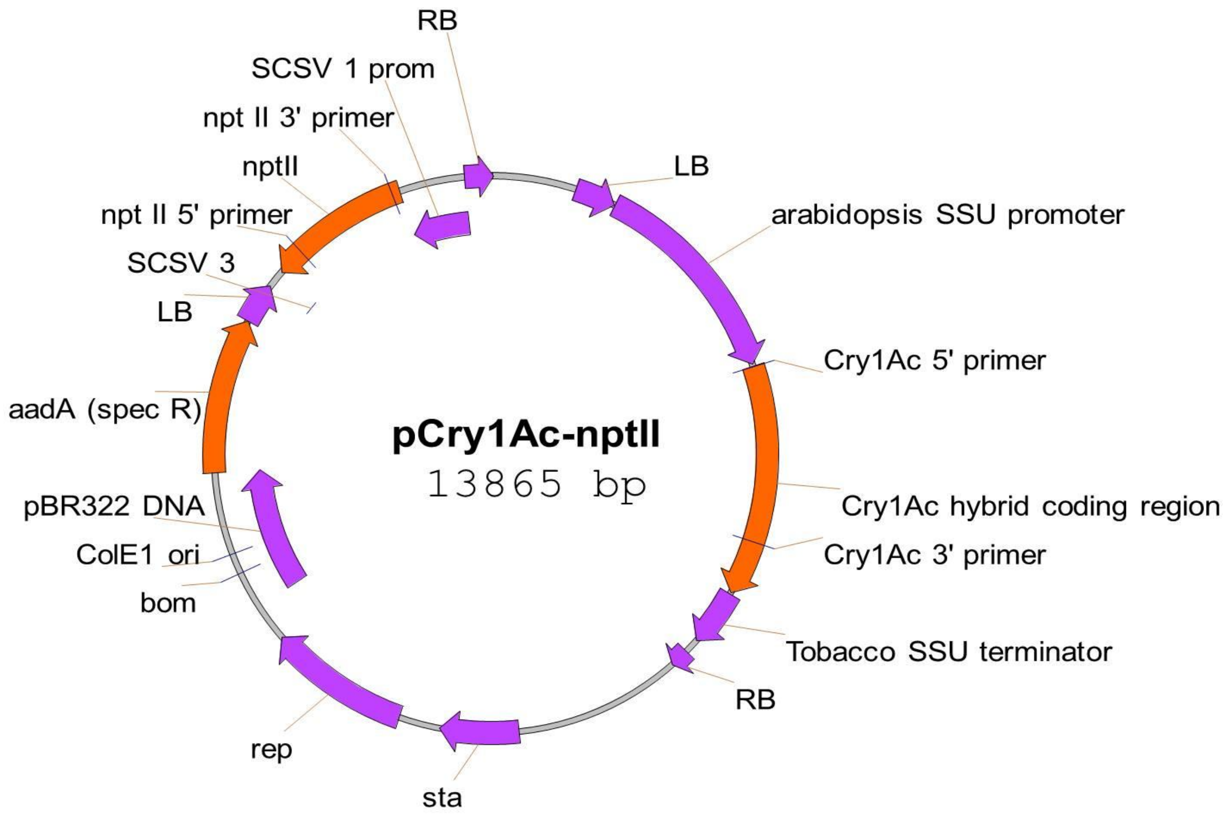

SUPPLEMENTARY FIGURE 2 *cry1Ac* expressing cultivated chickpea backcross populations grown under contained conditions during 2017-18. **(A)** BC_1F_3 derived from Cross B (L552 × BS 100E). **(B)** Closer view of BC_1F_3 plant. **(C)** Non-transgenic recipient parent L552. **(D)** Transgenic donor parent BS 100E. **(E)** BC_2F_2 derived from Cross C (PBG7 × BS 100E). **(F)** Closer view of BC_2F_2 plant. **(G)** Non-transgenic recipient parent PBG7.

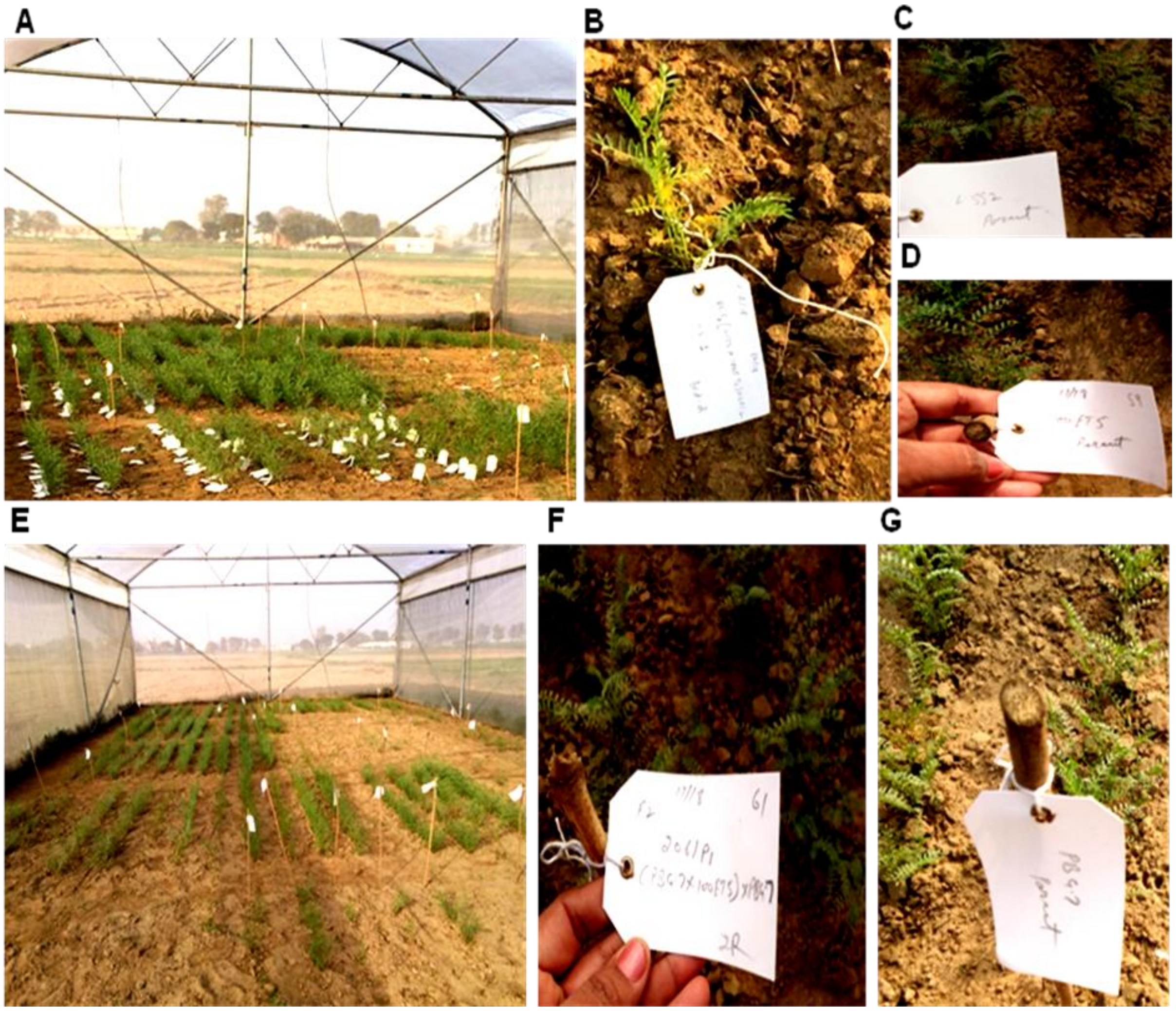

SUPPLEMENTARY FIGURE 3 Foreground selection of BC_1F_1 population derived from Cross A (PBG7 × BS 100B) through PCR using *cry1Ac* specific primers. P_1 indicates non-transgenic recipient parent PBG7; P_2 represents transgenic donor parent BS 100B; C refers to control PCR reaction without template DNA; the numbers 1 to 130 denote BC_1F_1 plants; M represents 50 bp DNA ladder (Cat. No. DM1100, Smobio Technology, Inc., Taiwan); forty six plants, namely 4, 5, 6, 7, 8, 14, 16, 17, 18, 21, 22, 24, 27, 28, 30, 35, 36, 42, 44, 52, 59, 60, 68, 72, 73, 77, 78, 81, 83, 84, 86, 87, 88, 89, 90, 93, 97, 100, 101, 102, 105, 106, 108, 119, 121 and 122 carried *cry1Ac* gene.

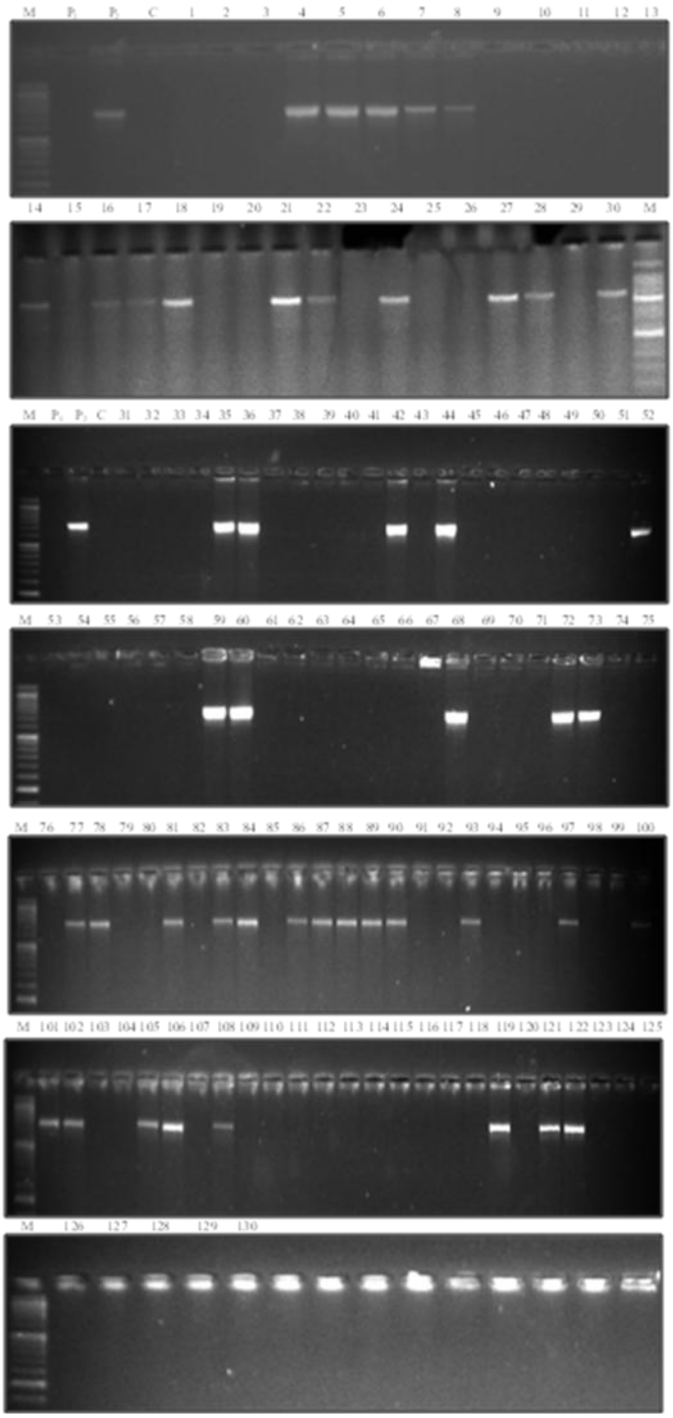

SUPPLEMENTARY FIGURE 4 Foreground selection of BC_1F_1 population derived from Cross B (L552 × BS 100E) through PCR using *cry1Ac* specific primers. P_1 indicates non-transgenic recipient parent L552; P_2 represents transgenic donor parent BS 100E; C refers to control PCR reaction without template DNA; the numbers 1 to 50 denote BC_1F_1 plants; M represents 50 bp DNA ladder (Cat. No. DM1100); twenty five plants, namely 1, 2, 4, 5, 7, 8, 10, 11, 12, 15, 24, 25, 28, 31, 33, 34, 36, 40, 41, 42, 44, 45, 46, 49 and 50 carried *cry1Ac* gene.

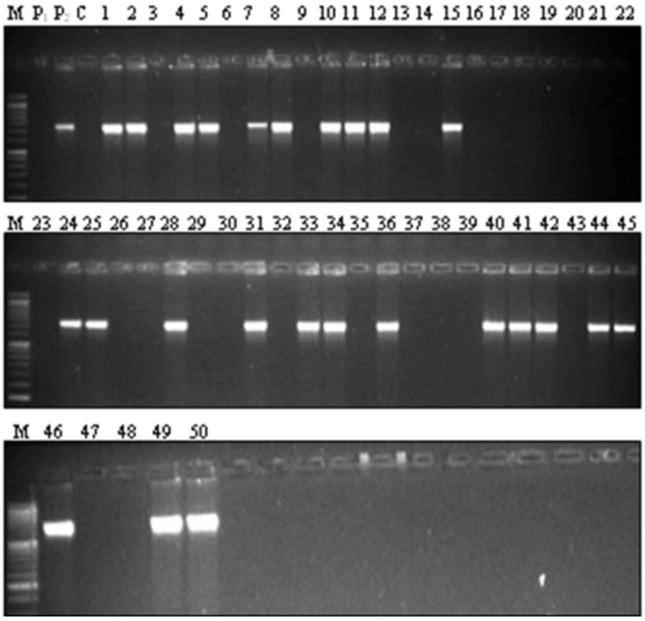

SUPPLEMENTARY FIGURE 5 Bioassay of BC_1F_1 plant derived from Cross A (PBG7 × BS 100B) expressing Cry1Ac for toxicity to *H. armigera* through detached leaf method. **(A)** Twig from BC_1F_1 plant showing healthy leaflets and mortality of neonate larvae. Arrows point dead larvae. **(B)** Twig from non-transgenic recipient parent PBG7 showing damage on the leaflets and survival of larva.

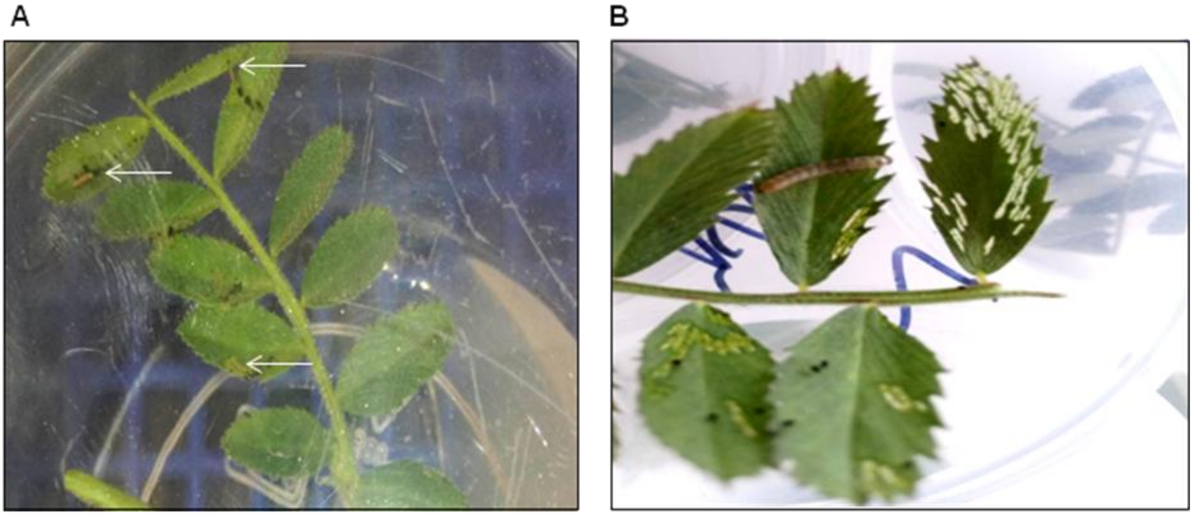

SUPPLEMENTARY FIGURE 6 Foreground selection of BC_1F_2 population derived from Cross A (PBG7 × BS 100B) through PCR using *cry1Ac* specific primers. P_1 indicates non-transgenic recipient parent PBG7; P_2 represents transgenic donor parent BS 100B; C refers to control PCR reaction without template DNA; the numbers 1 to 190 denote BC_1F_2 plants; M represents 50 bp DNA ladder (Cat. No. DM1100); sixteen plants, namely 6, 8, 12, 17, 18, 38, 40, 45, 47, 51, 53, 58, 88, 89, 90 and 94 carried *cry1Ac* gene.

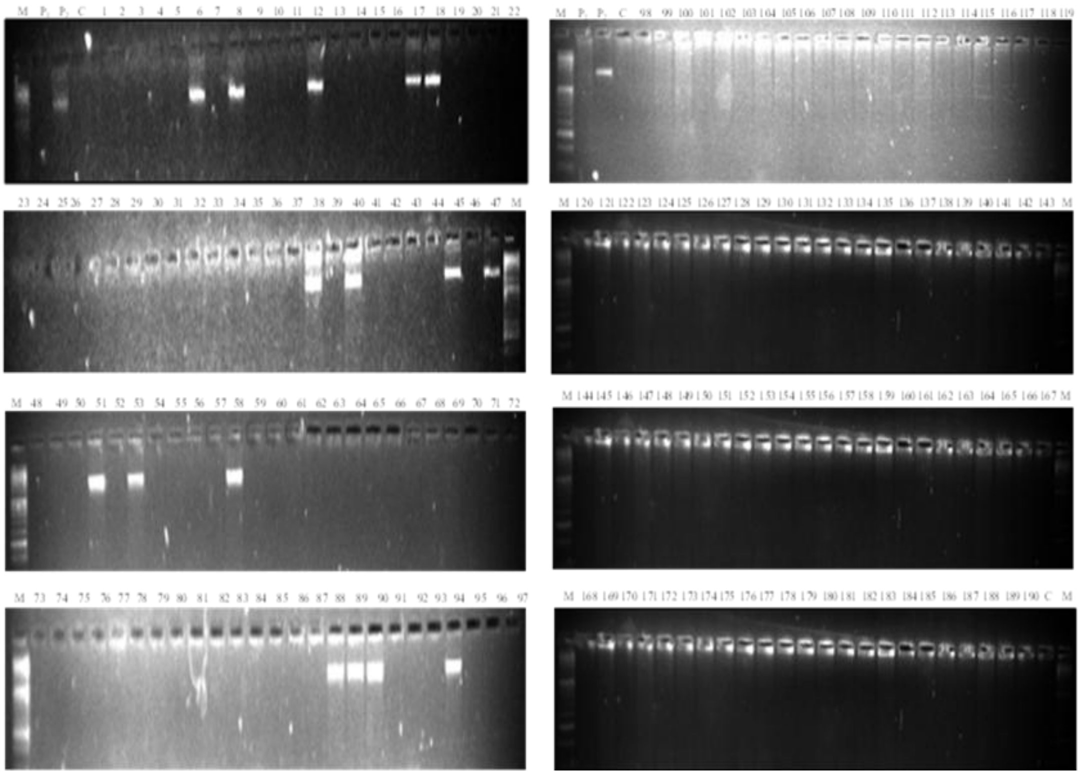

SUPPLEMENTARY FIGURE 7 Foreground selection of BC_1F_2 population derived from Cross B (L552 × BS 100E) through PCR using *cry1Ac* specific primers. P_1 refers non-transgenic recipient parent L552; P_2 indicates transgenic donor parent BS 100E; C refers to control; the numbers 1 to 17 represent BC_1F_2 plants; M denotes 50 bp DNA ladder (Cat. No. DM1100); thirteen plants, namely 1, 2, 3, 6, 8, 9, 10, 12, 13, 14, 15, 16 and 17 carried *cry1Ac* gene.

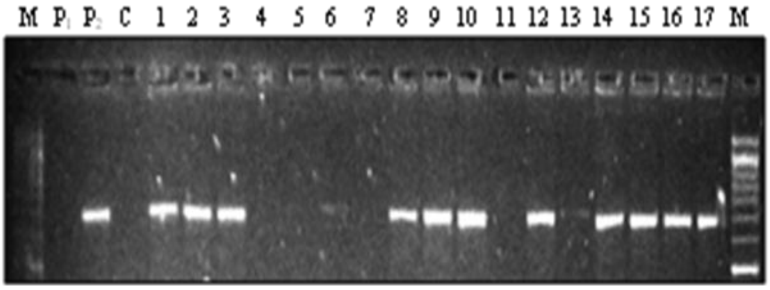

SUPPLEMENTARY FIGURE 8 Assessment of parental polymorphism through PCR using SSR markers. Lanes 1, 2 showing polymorphism using GA 6 marker; lanes 3, 4 displaying polymorphism with TA 59; lanes 5, 6 revealing polymorphism by GA 20 marker; lanes 7, 8 exhibiting polymorphism with GAA 40 marker; lanes 9, 10 displaying polymorphism with GAA 41 marker; lanes 11, 12 revealing polymorphism by TA 146 marker; lanes 13, 14 exhibiting polymorphism with CGMM 008 marker; lanes 15, 16 showing polymorphism using CGMM 016 marker; lanes 17, 18 displaying polymorphism by CGMM 022 marker; lanes 19, 20 revealing polymorphism with TA 34 marker; lanes 21, 22 exhibiting polymorphism using TA 64 marker; lanes 23, 24 displaying polymorphism with TAASH marker. P_1 indicates non-transgenic recipient parent PBG7; P_2 represents transgenic donor parent BS 100E.

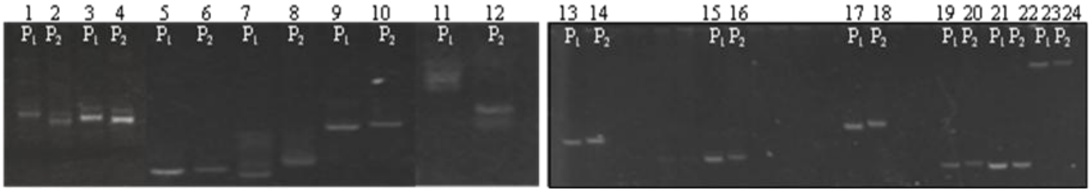

SUPPLEMENTARY TABLE 1 Introgression of *cry1Ac* from pod borer resistant transgenic chickpea lines into two elite commercial cultivars through marker assisted backcross breeding.

Year	Activity	Parent/Cross (Female × Male)	Seeds sown (Number)	Plants obtained (Number)	Population analyzed for					Designation of harvested seeds
					FS	ELISA	BS	P	IB	
2013-14	Seed multiplication	Male parent BS 100B	15	10	×	×	×	×	×	Transgenic donor parents
		Male parent BS 100E	15	11	×	×	×	×	×	
		Female parent PBG7	15	14	×	×	×	×	×	Non-transgenic recipient parents
		Female parent L552	15	13	×	×	×	×	×	
	Generation of crosses	PBG7 × BS 100B (55)	-	-	×	×	×	×	×	Cross A-F_1
		L552 × BS 100E (35)	-	-	×	×	×	×	×	Cross B-F_1
		PBG7 × BS 100E (35)	-	-	×	×	×	×	×	Cross C-F_1
2014-15	Raising of F_1 plants	Cross A-F_1	13	7	×	×	×	×	√ [7]	Cross A-F_2
		Cross B-F_1	10	7	×	×	×	×	√ [7]	Cross B-F_2
		Cross C-F_1	6	3	×	×	×	×	√ [3]	Cross C-F_2
	Generation of 1st backcross	Cross A-F_1 × PBG7 (250)	-	-	×	×	×	×	×	Cross A-BC_1F_1
		Cross B-F_1 × L552 (125)	-	-	×	×	×	×	×	Cross B-BC_1F_1
		Cross C-F_1 × PBG7 (75)	-	-	×	×	×	×	×	Cross C-BC_1F_1
2015-16	Raising of BC_1F_1	Cross A-BC_1F_1	150	130	√ [46]	√ [13]	×	√ [13]	√ [13]	Cross A-BC_1F_2
		Cross B-BC_1F_1	55	50	√ [25]	√ [9]	×	√ [9]	√ [7]	Cross B-BC_1F_2
		Cross C-BC_1F_1	30	18	×	×	×	√ [5]	√ [4]	Cross C-BC_1F_2
	Generation of 2nd backcross	Cross C-BC_1F_1 × PBG7 (70)	-	-	×	×	×	×	×	Cross C-BC_2F_1
2016-17	Raising of BC_1F_2	Cross A-BC_1F_2	280	190	√ [16]	×	×	×	×	Cross A-BC_1F_3
		Cross B-BC_1F_2	30	17	√ [13]	×	×	√ [9]	√ [9]	Cross B-BC_1F_3
	Raising of BC_2F_1	Cross C-BC_2F_1	31	31	×	×	×	×	×	Cross C-BC_2F_2
2017-18	Raising of BC_1F_3	Cross A-BC_1F_3	210	201	×	×	×	×	×	Cross A-BC_1F_4
		Cross B-BC_1F_3	27	26	×	×	×	√ [6]	√ [2]	Cross B-BC_1F_4
	Raising of BC_2F_2	Cross C-BC_2F_2	120	83	√ [10]	×	√ [10]	√ [7]$^{\Delta}$	√ [5]	Cross C-BC_2F_3
2018-19	Raising of BC_2F_3	Cross C-BC_2F_3	128	128	√ [106]	×	×	√ [12]$^{\Delta}$	√ [9]	Cross C-BC_2F_4

FS, Foreground Selection; ELISA, Enzyme Linked Immunosorbent Assay; BS, Background Selection; P, Phenotype; IB, Insect Bioassay; figures in round brackets are number of pollinations attempted. Figures in square brackets are number of plants identified after analysis; √, Yes; ×, No; $^{\Delta}$ Represents plants identified on basis of agronomic traits.

SUPPLEMENTARY TABLE 2 Estimation of Cry1Ac concentration in leaf tissues of BC_1F_1 populations derived from Cross A (PBG7 × BS 100B) and Cross B (L552 × BS 100E) through ELISA.

Step 1: Optical density values of positive calibrators and leaf tissue samples

**Cry1Ac calibrator/Leaf tissue sample**	**Optical density**		
	**R_1**	**R_2**	**R_3**
Negative control (NC)	0.05	0.05	0.05
1.5 ppb Cry1Ac calibrator (C1)	0.45	0.45	0.45
10 ppb Cry1Ac calibrator (C2)	1.95	1.98	1.96
25 ppb Cry1Ac calibrator (C3)	3.50	3.56	3.53
**BC_1F_1 plant number (derived from Cross A)**			
4	3.98	3.99	3.99
6	3.99	3.99	3.99
7	3.86	3.86	3.86
16	3.98	3.98	3.98
17	3.99	3.99	3.99
18	3.99	3.99	3.99
21	3.95	3.96	3.96
22	3.86	3.89	3.87
24	3.97	3.96	3.97
77	3.97	3.97	3.97
81	3.88	3.88	3.88
89	3.97	3.97	3.97
90	3.79	3.78	3.79
BS 100B	3.89	3.88	3.88
PBG7	0.05	0.05	0.05
**BC_1F_1 plant number (derived from Cross B)**			
1	3.96	3.96	3.96
2	3.95	3.95	3.95
4	3.93	3.93	3.93
25	3.93	3.93	3.93
34	3.98	3.98	3.98
36	3.96	3.96	3.96
41	3.98	3.98	3.98
42	3.93	3.93	3.93
45	3.94	3.93	3.94
BS 100E	3.98	3.96	3.97
L552	0.05	0.05	0.05

Step 2: Calculate subtracted values (calculate mean OD of NC and subtract it from OD of each calibrator and leaf tissue sample)

**Cry1Ac calibrator/Leaf tissue sample**	**Subtracted values**		
	**R_1**	**R_2**	**R_3**
NC	0	0	0
C1	0.40	0.40	0.40
C2	1.90	1.93	1.91
C3	3.45	3.51	3.48
**BC_1F_1 plant number (derived from Cross A)**			
4	3.93	3.94	3.94
6	3.94	3.94	3.94
7	3.81	3.81	3.81
16	3.93	3.93	3.93
17	3.94	3.94	3.94
18	3.94	3.94	3.94
21	3.90	3.91	3.91
22	3.81	3.84	3.82
24	3.92	3.91	3.92
77	3.92	3.92	3.92
81	3.83	3.83	3.83
89	3.92	3.92	3.92
90	3.74	3.73	3.74
BS 100B	3.84	3.83	3.83
PBG7	0	0	0
**BC_1F_1 plant number (derived from Cross B)**			
1	3.91	3.91	3.91
2	3.90	3.90	3.90
4	3.88	3.88	3.88
25	3.88	3.88	3.88
34	3.93	3.93	3.93
36	3.91	3.91	3.91
41	3.93	3.93	3.93
42	3.88	3.88	3.88
45	3.89	3.88	3.89
BS 100E	3.93	3.91	3.92
L552	0	0	0

Step 3: Generation of linear scale graph of mean OD of each calibrator against its Cry1Ac concentration (first calculate mean OD of each calibrator C1, C2 and C3; then generate ‘y’ equation and R^2 based on mean ODs of calibrators; thereafter insert scatter chart in excel sheet)

**Cry1Ac concentration in ppb (x value)**	**Mean OD (y value)**
1.5 (C1)	0.45
10 (C2)	1.96
25 (C3)	3.53

To generate ‘y’ equation (y = mx + b, where m = slope and b = y intercept), select ‘x’ and ‘y’ values given in Step 3. To generate linear scale graph, insert scatter chart in Microsoft Excel sheet:

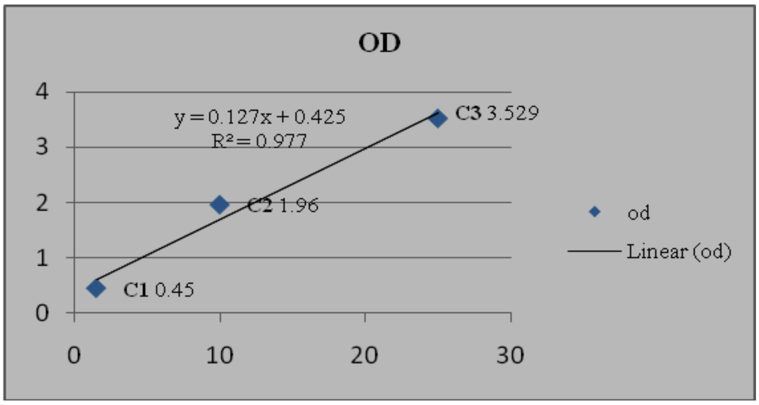

Mean ODs of calibrators (y axis)

Cry1Ac concentration in ppb (x axis)

Step 4: Estimation of Cry1Ac concentration of each calibrator and leaf tissue sample was done as follows: OD value of calibrator or leaf tissue sample (value as per Step 2) - 0.425/ 0.127

**Cry1Ac calibrator/ Leaf tissue sample**	**Cry1Ac concentration (ppb)**		
	**R_1**	**R_2**	**R_3**
NC	0	0	0
C1	0	0	0
C2	11.61	11.85	11.69
C3	23.82	24.29	24.05
**BC_1F_1 plant number (derived from Cross A)**			
4	27.60	27.68	27.68
6	27.68	27.68	27.68
7	26.65	26.658	26.65
16	27.60	27.60	27.60
17	27.68	27.68	27.68
18	27.68	27.68	27.68
21	27.36	27.44	27.44
22	26.65	26.89	26.74
24	27.52	27.44	27.52
77	27.52	27.52	27.52
81	26.81	26.81	26.81
89	27.52	27.52	27.52
90	26.10	26.02	26.10
BS 100B	26.89	26.81	26.81
PBG7	0	0	0
**BC_1F_1 plant number (derived from Cross B)**			
1	27.44	27.44	27.44
2	27.36	27.36	27.36
4	27.20	27.20	27.20
25	27.20	27.20	27.20
34	27.60	27.60	27.60
36	27.44	27.44	27.44
41	27.60	27.60	27.60
42	27.20	27.20	27.20
45	27.28	27.20	27.28
BS 100E	27.60	27.44	27.52
L552	0	0	0

Step 5: Estimation of Cry1Ac concentration in ppm or µg g^{-1} by applying the formula: OD value (as per Step 4) × dilution factor 1 (38.46) × dilution factor 2 (11, as samples were diluted at 1:11)/ 1000

**Cry1Ac calibrator/Leaf tissue sample**	**Cry1Ac concentration (µg g^{-1} leaf tissue)**		
	**R_1**	**R_2**	**R_3**
NC	0	0	0
C1	0	0	0
C2	4.91	5.01	4.94
C3	10.08	10.28	10.17
**BC_1F_1 plant number (derived from Cross A)**			
4	11.68	11.71	11.71
6	11.71	11.71	11.71
7	11.27	11.28	11.27
16	11.68	11.68	11.68
17	11.71	11.71	11.71
18	11.71	11.71	11.71
21	11.57	11.61	11.61
22	11.27	11.38	11.31
24	11.64	11.61	11.64
77	11.64	11.64	11.64
81	11.34	11.34	11.34
89	11.64	11.64	11.64
90	11.04	11.01	11.04
BS 100B	11.38	11.34	11.34
PBG7	0	0	0
**BC_1F_1 plant number (derived from Cross B)**			
1	11.61	11.61	11.61
2	11.57	11.57	11.57
4	11.51	11.51	11.51
25	11.51	11.51	11.51
34	11.68	11.68	11.68
36	11.61	11.61	11.61
41	11.68	11.68	11.68
42	11.51	11.51	11.51
45	11.54	11.51	11.54
BS 100E	11.68	11.61	11.64
L552	0	0	0

SUPPLEMENTARY TABLE 3 SSR markers used to analyze parental polymorphism and carry out background selection of BC_2F_2 plants derived from Cross C (PBG7 × BS 100E).

S. No.	Marker name	Status of marker	Reference	Chromosomal location	Reference for chromosomal location	PIC value	Reference for PIC value
1	CGMM 001*	P	Gujaria et al. (2011)	8	https://plantgarden.jp>list>CAPS	NA	
2	CGMM 002	M	Gujaria et al. (2011)				
3	CGMM 003	M	Gujaria et al. (2011)				
4	CGMM 004	M	Gujaria et al. (2011)				
5	CGMM 005	M	Gujaria et al. (2011)				
6	CGMM 006	M	Gujaria et al. (2011)				
7	CGMM 007	M	Gujaria et al. (2011)				
8	**CGMM 008**	P	Gujaria et al. (2011)	NA	https://plantgarden.jp>list>CAPS	NA	-
9	CGMM 009	M	Gujaria et al. (2011)				
10	CGMM 010	M	Gujaria et al. (2011)				
11	CGMM 011	M	Gujaria et al. (2011)				
12	CGMM 012*	P	Gujaria et al. (2011)	2	Gujaria et al. (2011)	NA	
13	CGMM 013	M	Gujaria et al. (2011)				
14	CGMM 014	M	Gujaria et al. (2011)				
15	CGMM 015	M	Gujaria et al. (2011)				
16	**CGMM 016**	P	Gujaria et al. (2011)	3	https://plantgarden.jp>list>CAPS	NA	-
17	CGMM 017	M	Gujaria et al. (2011)				
18	CGMM 018	M	Gujaria et al. (2011)				
19	CGMM 020	M	Gujaria et al. (2011)				
20	CGMM 021	M	Gujaria et al. (2011)				
21	**CGMM 022**	P	Gujaria et al. (2011)	5	https://plantgarden.jp>list>CAPS	NA	-
22	CGMM 023	M	Gujaria et al. (2011)				
23	CGMM 024	M	Gujaria et al. (2011)				
24	CGMM 025	M	Gujaria et al. (2011)				
25	CGMM 026	M	Gujaria et al. (2011)				
26	CGMM 027	M	Gujaria et al. (2011)				
27	CGMM 028	M	Gujaria et al. (2011)				
28	CGMM 029	M	Gujaria et al. (2011)				
29	CGMM 030	M	Gujaria et al. (2011)				
30	CGMM 031	M	Gujaria et al. (2011)				
31	CGMM 032	M	Gujaria et al. (2011)				
32	CGMM 033	M	Gujaria et al. (2011)				
33	CGMM 062	M	Gujaria et al. (2011)				
34	CGMM 063	M	Gujaria et al. (2011)				
35	CGMM 064	M	Gujaria et al. (2011)				
36	CGMM 065	M	Gujaria et al. (2011)				
37	CGMM 066	M	Gujaria et al. (2011)				
38	CGMM 067	M	Gujaria et al. (2011)				
39	CGMM 068	M	Gujaria et al. (2011)				
40	CGMM 069	M	Gujaria et al. (2011)				
41	CGMM 070	M	Gujaria et al. (2011)				
42	CGMM 071	M	Gujaria et al. (2011)				
43	CGMM 072	M	Gujaria et al. (2011)				
44	CGMM 073	M	Gujaria et al. (2011)				
45	CGMM 074	M	Gujaria et al. (2011)				
46	CGMM 075	M	Gujaria et al. (2011)				
47	CGMM 076	M	Gujaria et al. (2011)				
48	CGMM 077	M	Gujaria et al. (2011)				
49	CGMM 078	M	Gujaria et al. (2011)				
50	CGMM 138	M	Gujaria et al. (2011)				
51	CaM 0038	M	Thudi et al. (2011)				
52	CaM 0046	M	Thudi et al. (2011)				
53	CaM 0244	M	Thudi et al. (2011)				
54	CaM 0594	M	Thudi et al. (2011)				
55	CaM 0805	M	Thudi et al. (2011)				
56	CaM 1101	M	Thudi et al. (2011)				
57	CaM 1125*	P	Thudi et al. (2011)	6	Thudi et al. (2011)	NA	
58	CaM 1402	M	Thudi et al. (2011)				
59	CaM 1502	M	Thudi et al. (2011)				
60	CaM 1903*	P	Thudi et al. (2011)	4	Nayak (2010)	0.52	Sachdeva et al. (2018)
61	CaM 2049	M	Thudi et al. (2011)				
62	GA 2	M	Winter et al. (1992)				
63	GA 4	M	Winter et al. (1992)				
64	**GA 6**	P	Winter et al. (1992)	8	Millan et al. (2010)	0.47	Sachdeva et al. (2018)
65	GA 8*	P	Winter et al. (1992)	NA		0.24	Vashist et al. (2019)
66	GA 9	M	Winter et al. (1992)				
67	GA 11	M	Winter et al. (1992)				
68	GA 13	M	Winter et al. (1992)				
69	GA 14	M	Winter et al. (1992)				
70	GA 17	M	Winter et al. (1992)				
71	**GA 20**	P	Winter et al. (1992)	2	Millan et al. (2010)	0.49	Ghaffari et al. (2014)
72	GA 21	M	Winter et al. (1992)				

73	GA 22	M	Winter et al. (1992)				
74	GA 26*	P	Winter et al. (1992)	6	Winter et al. (2000)	0.61	Jha et al. (2018)
75	GA 31	M	Winter et al. (1992)				
76	GA 33	M	Winter et al. (1992)				
77	GA 105	M	Winter et al. (1992)				
78	GA 108	M	Winter et al. (1992)				
79	GA 117	M	Winter et al. (1992)				
80	GA 137	M	Winter et al. (1992)				
81	GAA 39*	P	Winter et al. (1992)	6	Nayak (2010)	0.30	Vashist et al. (2019)
82	**GAA 40**	P	Winter et al. (1992)	1	Winter et al. (2000)	0.37	Sefera et al. (2011)
83	**GAA 41**	P	Winter et al. (1992)	6	Nayak et al. (2010)	0.82	Ganguly et al. (2008)
84	GAA 42	M	Winter et al. (1992)				
85	GAA 43	M	Winter et al. (1992)				
86	GAA 44	M	Winter et al. (1992)				
87	GAA 45*	P	Winter et al. (1992)	3	Jadhav et al. (2015)	0.03	Jadhav et al. (2015)
88	GAA 46	M	Winter et al. (1992)				
89	GAA 50	M	Winter et al. (1992)				
90	GAA 51	M	Winter et al. (1992)				
91	GAA 54	M	Winter et al. (1992)				
92	GAA 58	M	Winter et al. (1992)				
93	GAA 60	M	Winter et al. (1992)				
94	GAA 129b	M	Winter et al. (1992)				
95	TA 2	M	Winter et al. (1992)				
96	TA 18	M	Winter et al. (1992)				
97	TA 20	M	Winter et al. (1992)				
98	TA 25	M	Winter et al. (1992)				
99	TA 28	M	Winter et al. (1992)				
100	**TA 34**	P	Winter et al. (1992)	3	Winter et al. (2000)	0.39	Choudhary et al. (2012)
101	TA 36	M	Winter et al. (1992)				
102	TA 37	M	Winter et al. (1992)				
103	TA 43	M	Winter et al. (1992)				
104	TA 44	M	Winter et al. (1992)				
105	TA 45	M	Winter et al. (1992)				
106	TA 46	M	Winter et al. (1992)				
107	TA 47	M	Winter et al. (1992)				
108	TA 53	M	Winter et al. (1992)				
109	**TA 59**	P	Winter et al. (1992)	2	Winter et al. (2000)	0.36	Choudhary et al. (2012)
110	**TA 64**	P	Winter et al. (1992)	3	Winter et al. (2000)	0.34	Choudhary et al. (2012)
111	TA 66	M	Winter et al. (1992)				
112	TA 71	M	Winter et al. (1992)				
113	TA 72	M	Winter et al. (1992)				
114	TA 76s*	P	Winter et al. (1992)	NA	Sefera et al. (2011)	0.57	Rizvi et al. (2013)
115	TA 78	M	Winter et al. (1992)				
116	TA 80	M	Winter et al. (1992)				
117	TA 87	M	Winter et al. (1992)				
118	TA 89	M	Winter et al. (1992)				
119	TA 93	M	Winter et al. (1992)				
120	TA 103	M	Winter et al. (1992)				
121	TA 104	M	Winter et al. (1992)				
122	TA 106	M	Winter et al. (1992)				
123	TA 108	M	Winter et al. (1992)				
124	TA 113	M	Winter et al. (1992)				
125	TA 114	M	Winter et al. (1992)				
126	TA 116	M	Winter et al. (1992)				
127	TA 117	M	Winter et al. (1992)				
128	TA 118	M	Millan et al. (2010)				
129	TA 125	M	Winter et al. (1992)				
130	TA 127	M	Winter et al. (1992)				
131	TA 135	M	Winter et al. (1992)				
132	TA 136	M	Winter et al. (1992)				
133	TA 140	M	Winter et al. (1992)				
134	TA 141	M	Winter et al. (1992)				
135	TA 142	M	Winter et al. (1992)				
136	TA 144	M	Winter et al. (1992)				
137	**TA 146**	P	Winter et al. (1992)	4	Winter et al. (2000)	0.72	Choudhary et al. (2012)
138	TA 159	M	Millan et al. (2010)				
139	TA 167	M	Winter et al. (1992)				
140	TA 176	M	Winter et al. (1992)				
141	TA 179	M	Winter et al. (1992)				
142	TA 180	M	Winter et al. (1992)				
143	TA 186	M	Winter et al. (1992)				
144	TA 198	M	Winter et al. (1992)				
145	TA 200	M	Winter et al. (1992)				
146	TA 203	M	Winter et al. (1992)				
147	TA 206	M	Winter et al. (1992)				
148	TAA 55	M	Winter et al. (1992)				
149	TAA 57	M	Winter et al. (1992)				
150	TAA 58	M	Winter et al. (1992)				

151	TAA 61*	P	Winter et al. (1992)	NA		NA	
152	TAA 104	M	Winter et al. (1992)				
153	TAA 107	M	Winter et al. (1992)				
154	TAA 137	M	Winter et al. (1992)				
155	TAA 169	M	Winter et al. (1992)				
156	TAA 194	M	Winter et al. (1992)				
157	**TAASH**	P	Winter et al. (1992)	5	Winter et al. (2000)	0.86	Joshi et al. (2013)
158	TS 5	M	Winter et al. (1992)				
159	TS 10	M	Winter et al. (1992)				
160	TS 11	M	Winter et al. (1992)				
161	TS 12	M	Winter et al. (1992)				
162	TS 16*	P	Winter et al. (1992)	NA		NA	
163	TS 19	M	Winter et al. (1992)				
164	TS 23	M	Winter et al. (1992)				
165	TS 24	M	Winter et al. (1992)				
166	TS 29	M	Winter et al. (1992)				
167	TS 35	M	Winter et al. (1992)				
168	TS 39	M	Winter et al. (1992)				
169	TS 43	M	Winter et al. (1992)				
170	TS 45*	P	Winter et al. (1992)	8	Sefera et al. (2011)	0.66	Sefera et al. (2011)
171	TS 47	M	Winter et al. (1992)				
172	TS 52	M	Winter et al. (1992)				
173	TS 53	M	Winter et al. (1992)				
174	TS 54*	P	Winter et al. (1992)	4	Nayak (2010)	0.81	Winter et al. (2000)
175	TS 58	M	Winter et al. (1992)				
176	TS 72	M	Winter et al. (1992)				
177	TS 74	M	Winter et al. (1992)				
178	TS 79	M	Winter et al. (1992)				
179	TS 83	M	Winter et al. (1992)				
180	TS 84	M	Winter et al. (1992)				
181	TS 104	M	Winter et al. (1992)				
182	TR 1	M	Winter et al. (1992)				
183	TR 2	M	Winter et al. (1992)				
184	TR 3	M	Winter et al. (1992)				
185	TR 7	M	Winter et al. (1992)				
186	TR 8	M	Winter et al. (1992)				
187	TR 20	M	Winter et al. (1992)				
188	TR 26	M	Winter et al. (1992)				
189	TR 29	M	Winter et al. (1992)				
190	TR 31	M	Winter et al. (1992)				
191	TR 32	M	Winter et al. (1992)				
192	TR 33	M	Winter et al. (1992)				
193	TR 35	M	Winter et al. (1992)				
194	TR 40	M	Winter et al. (1992)				
195	TR 43	M	Winter et al. (1992)				
196	TR 44	M	Winter et al. (1992)				
197	TR 45	M	Winter et al. (1992)				
198	TR 55	M	Winter et al. (1992)				
199	TR 56	M	Winter et al. (1992)				
200	TR 59	M	Winter et al. (1992)				
201	TR 60	M	Winter et al. (1992)				
202	NCPGR 21	M	Sethy et al. (2006)				
203	NCPGR 127	M	Varshney et al. (2014)				
204	NCPGR 141	M	Varshney et al. (2014)				
205	NCPGR 171	M	Varshney et al. (2014)				
206	NCPGR 247	M	Varshney et al. (2014)				
207	CaSTMS 11	M	Hüttel et al. (1999)				
208	H1I16	M	Lichtenzveig et al. (2005)				
209	H1A19	M	Lichtenzveig et al. (2005)				
210	H4G11	M	Lichtenzveig et al. (2005)				

M, Monomorphic marker; P, Polymorphic marker; markers in bold depict polymorphic markers that had high reproducibility and were used to carry out background selection; *represents polymorphic markers that had low reproducibility; NA, Not Available.

Jadhav, A. A., Rayate, S. J., Mhase, L. B., Thudi, M., Chitikineni, A., Harer, P. N., Jadhav, A. S., Varshney, R., and Kulwal, P. (2015). Marker-trait association study for protein content in chickpea (*Cicer arietinum* L.). *J. Genet.* 94, https://doi.org/10.1007/s12041-015-0529-6.

Choudhary, S., Kaur, J., Chhuneja, P., Sandhu, J. S., Singh, I., Singh, S., and Sirari, A. (2012). Assessment of genetic diversity in *kabuli* chickpea (*Cicer arietinum* L.) genotypes in relation to seed size using SSR markers. *Journal of Food Legumes* 26, 1–4.

Ganguly, A. K., Chawla, G., Yadav, R., and Kumar, R. (2008). STMS profiling of chickpea (*Cicer arietinum*) with regards to nematode resistance. *Indian J. Nematol.* 38, 209–217.

Ghaffari, P., Talebi, R., and Keshavarzi, F. (2014). Genetic diversity and geographical differentiation of Iranian landrace, cultivars, and exotic chickpea lines as revealed by morphological and microsatellite markers. *Physiol. Mol. Biol. Plants* 20, 225–233.

Gujaria, N., Kumar, A., Dauthal, P., Dubey, A., Hiremath, P., Prakash, A. B., Farmer, A., Bhide, M., Shah, T., Gaur, P. M., Upadhyaya, H. D., Bhatia, S., Cook, D. R., May, G. D., and Varshney, R. K. (2011). Development and use of genic molecular markers (GMMs) for construction of a transcript map of chickpea (*Cicer arietinum* L.). *Theor. Appl. Genet.* 122, 1577–1589.

Rizvi, H., Babu, B. K., and Agrawal, P. K. (2013). Molecular analysis of *kabuli* and *desi* type of Indian chickpea (*Cicer arietinum* L.) cultivars using STMS markers. *J. Plant Biochem. Biotech.*, https://doi.org/10.1007/s13562-012-0187-1.

Hüttel, B., Winter, P., Weising, K., Choumane, W., Weigand, F., and Kahl, G. (1999). Sequence-tagged microsatellite site markers for chickpea (*Cicer arietinum* L.). *Genome* 42, 210–217.

Lichtenzveig, J., Scheuring, C., Dodge, J., Abbo, S., and Zhang, H. B. (2005). Construction of BAC and BIBAC libraries and their applications for generation of SSR markers for genome analysis of chickpea, *Cicer arietinum*L. *Theor. Appl. Genet.*110, 492–510.

Millan, T., Winter, P., Jüngling, R., Gil, J., Rubio, J., Cho, S., Cobos, M. J., Iruela, M., Rajesh, P. N., Tekeoglu, M., Kahl, G., and Muehlbauer, F. J. (2010). A consensus genetic map of chickpea (*Cicer arietinum* L.) based on 10 mapping populations. *Euphytica* 175, 175–189.

Sethy, N. K., Shokeen, B., Edwards, K. J., and Bhatia, S. (2006). Development of microsatellite markers and analysis of intraspecific genetic variability in chickpea (*Cicer arietinum* L.). *Theor. Appl. Genet.* 112, 1416–1428.

Nayak, S. N. (2010). Identification of QTLs and genes for drought tolerance using linkage mapping and association mapping approaches in chickpea (*Cicer arietinum* L.). Ph.D. thesis, Osmania University, Hyderabad, India.

Nayak, S. N., Zhu, H., Varghese, N., Datta, S., Choi, H. K., Horres, R., Jüngling, R., Singh, J., Kishor, P. K., Sivaramakrishnan, S., Hoisington, D. A., Kahl, G., Winter, P., Cook, D. R., and Varshney, R. K. (2010). Integration of novel SSR and gene-based SNP marker loci in the chickpea genetic map and establishment of new anchor points with *Medicago truncatula* genome. *Theor. Appl. Genet.*120, 1415–1441.

Sachdeva, S., Bharadwaj, C., Sharma, V., Patil, B. S., Soren, K. R., Roorkiwal, M., Varshney, R. K., and Bhat, K. V. (2018). Molecular and phenotypic diversity among chickpea (*Cicer arietinum*) genotypes as a function of drought tolerance. *Crop & Pasture Sci.* 69, 142–153.

Sefera, T., Abebie, B., Gaur, P. M., Assefa, K., and Varshney, R. K. (2011). Characterisation and genetic diversity analysis of selected chickpea cultivars of nine countries using simple sequence repeat (SSR) markers. *Crop & Pasture Sci*. 62, 177–187.

Thudi, M., Bohra, A., Nayak, S. N., et al. (2011). Novel SSR markers from BAC-end sequences, DArT arrays and a comprehensive genetic map with 1,291 marker loci for chickpea (*Cicer arietinum*). *PLoS ONE* 6, e27275.

Jha, U. C., Jha, R., Bohra, A., Parida, S. K., Kole, P. C., Thakro, V., Singh, D., and Singh, N. P. (2018). Population structure and association analysis of heat stress relevant traits in chickpea (*Cicer arietinum* L.). *3 Biotech* 8, 43.

Varshney, R. K., Thudi, M., Nayak, S. N., et al. (2014) Genetic dissection of drought tolerance in chickpea (*Cicer arietinum* L.). *Theor. Appl. Genet.*127, 445–462.

Vashist, U., Boora, K. S., and Kumar, M. (2019). Evaluation of genetic diversity among chickpea (*Cicer arietinum* L.) genotypes using PCR based simple sequence repeats markers. *The Pharma Innovation Journal* 8, 182–188.

Winter, P., Benko-Iseppon, A. M., Hüttel, B., Ratnaparkhe, M., Tullu, A., Sonnante, G., Pfaff, T., Tekeoglu, M., Santra, D., Sant, V. J., Rajesh, P. N., Kahl, G., and Muehlbauer, F. J. (2000). A linkage map of the chickpea (*Cicer arietinum* L.) genome based on recombinant inbred lines from a *C. arietinum* × *C. reticulatum* cross: localization of resistance genes for *Fusarium* wilt races 4 and 5. *Theor. Appl. Genet.* 101, 1155–1163.

Winter, P., Pfaff, T., Udupa, S. M., Hüttel, B., Sharma, P. C., Sahi, S., Arreguin-Espinoza, R., Weigand, F., Muehlbauer, F. J., and Kahl, G. (1992). Characterization and mapping of sequence-tagged microsatellite sites in the chickpea (*C. arietinum* L.) genome. *Mol. Gen. Genet.* 262, 90–101.

Joshi, N., Sharma, S., Subramanian, R. B., and Rao, K. S. (2013). Genetic fingerprinting of chickpea (*Cicer arietinum* L.) germplasm using morphological and molecular markers. *Asian J. Exp. Biol. Sci.* 4, 398–405.

SUPPLEMENTARY TABLE 4 Recurrent parent genome recovery in *cry1Ac* positive BC_2F_2 plants derived from Cross C (PBG7 × BS 100E) using polymorphic SSR markers.

**Marker name**	**Plant number**									
	**1**	**2**	**8**	**9**	**12**	**20**	**26**	**33**	**39**	**44**
CGMM 008	-	A	A	A	A	A	A	A	-	A
CGMM 016	A	A	A	A	A	A	A	A	A	A
CGMM 022	A	A	A	A	A	A	A	A	A	A
GA 6	-	A	A	A	A	A	A	A	A	A
GA 20	A	A	A	A	A	A	A	A	A	A
GAA 40	A	-	A	A	-	-	-	-	-	-
GAA 41	A	A	A	A	A	A	A	A	A	A
TA 34	A	A	A	A	A	A	A	A	A	A
TA 59	A	-	A	A	A	A	-	A	A	A
TA 64	A	A	A	A	A	A	A	A	A	A
TA 146	A	-	A	A	A	A	-	A	A	A
TAASH	A	A	A	A	A	A	A	A	A	A
cry1Ac specific PCR primer	B	B	B	B	B	B	B	B	B	B
A (%)	90.9	90.0	92.3	92.3	91.7	91.7	90.0	91.7	90.9	91.7
Average recurrent parent genome recovery = 91.3 %										

'A' represents presence of allele corresponding to recurrent parent PBG7, 'B' denotes presence of allele for donor parent BS 100E.

SUPPLEMENTARY TABLE 5 Agronomic traits of BC_2F_2 and BC_2F_3 populations derived from Cross C (PBG7 × BS 100E).

S. No.	Plant number/ Parent	Agronomic trait										Recurrent parent phenome recovery (%)
		Days to 50 % flowering	Number of branches per plant	Days to maturity	Plant height (cm)	Number of pods per plant	Number of seeds per plant	100-seed weight (g)	Biological yield (g)	Seed yield per plant (g)	Harvest Index[€] (%)	
BC_2F_2												
1	1	84 (93.33)	13 (76.47)	149 (96.13)	51.3 (86.51)	44 (73.33)	80 (70.80)	15.6 (93.97)	40.22 (77.97)	13.21 (76.36)	32.84 (97.91)	84.28
2	2	89 (98.89)	15 (88.23)	153 (98.71)	55.5 (93.59)	55 (91.67)	102 (90.26)	15.5 (93.37)	47.16 (91.43)	15.45 (89.31)	32.76 (97.67)	93.31
3	8	87 (96.67)	16 (94.12)	152 (98.06)	57.5 (96.96)	46 (76.67)	85 (75.22)	16.2 (97.59)	44.36 (86.00)	14.12 (81.62)	31.83 (94.90)	89.78
4	9	88 (97.78)	12 (70.59)	154 (99.35)	58.1 (97.98)	43 (71.67)	78 (69.03)	15.8 (95.18)	38.34 (74.33)	12.43 (71.85)	32.42 (96.66)	84.44
5	12	85 (94.44)	12 (70.59)	151 (97.42)	50.2 (84.65)	49 (81.67)	94 (83.18)	15.6 (93.97)	43.78 (84.88)	14.32 (82.77)	32.71 (97.52)	87.11
6	20	89 (98.89)	15 (88.23)	153 (98.71)	58.3 (98.31)	53 (88.33)	98 (86.72)	15.1 (90.96)	49.56 (96.08)	15.20 (87.86)	30.67 (91.44)	92.55
7	26	89 (98.89)	14 (82.35)	154 (99.35)	57.6 (97.13)	59 (98.33)	112 (99.11)	15.2 (91.57)	51.00 (98.87)	16.86 (97.46)	33.06 (97.46)	96.05
8	33	86 (95.55)	16 (94.12)	150 (96.77)	51.4 (86.68)	54 (90.00)	99 (87.61)	15.3 (92.17)	47.62 (92.32)	15.23 (88.03)	31.98 (95.35)	91.86
9	39	87 (96.67)	15 (88.23)	151 (97.42)	55.6 (93.76)	57 (95.00)	108 (95.57)	15.3 (92.17)	50.50 (97.91)	16.38 (94.68)	32.43 (96.69)	94.81
10	44	86 (95.55)	15 (88.23)	151 (97.42)	56.2 (94.77)	58 (96.67)	108 (95.57)	15.3 (92.17)	51.02 (98.91)	16.60 (95.95)	32.54 (97.02)	95.23
11	BS 100E	82	8	145	48.4	16	28	13.3	23.30	4.13	17.72	-
12	PBG7	90	17	155	59.3	60	113	16.6	51.58	17.30	33.54	-
										Average recurrent parent phenome recovery = 90.94 %		
BC_2F_3												
1	2-1	88	14	151	57.5	51	98	15.2	41.14	13.45	32.69	
2	2-2	88	13	150	54.6	48	94	15.6	46.20	13.23	28.64	
3	2-3	89	15	151	55.0	53	101	15.3	45.67	15.28	33.46	
	Mean ± SD	88.33 ± 0.58	14.00 ± 1.00	150.67 ± 0.58	55.70 ± 1.58	50.67 ± 2.52	97.67 ± 3.51	15.36 ± 0.21	44.34 ± 2.78	13.99 ± 1.12	31.59 ± 2.59	
4	8-1	85	15	151	55.5	42	77	16.0	43.66	14.42	33.05	
5	8-2	87	16	153	58.6	45	86	15.7	41.20	14.10	34.22	
6	8-3	86	17	151	53.3	46	87	15.8	47.35	14.32	30.24	
	Mean ± SD	86.00 ± 1.00	16.00 ± 1.00	151.67 ± 1.15	55.80 ± 2.66	44.33 ± 2.08	83.33 ± 5.51	15.83 ± 0.15	44.07 ± 3.09	14.28 ± 0.16	32.50 ± 2.04	
7	20-1	88	16	155	55.3	54	99	15.6	50.56	15.20	30.06	
8	20-2	86	15	154	57.4	55	100	14.8	51.23	15.51	30.27	
9	20-3	88	16	154	59.7	52	98	15.7	54.45	16.20	29.75	
	Mean ± SD	87.33 ± 1.15	15.67 ± 0.58	154.30 ± 0.58	57.47 ± 2.20	53.67 ± 1.53	99.00 ± 1.00	15.37 ± 0.49	52.08 ± 2.08	15.64 ± 0.51	30.03 ± 0.26	
10	26-1	88	14	152	55.6	57	116	15.5	55.58	17.25	31.04	
11	26-2	89	13	151	58.5	51	107	15.1	52.34	16.45	31.43	
12	26-3	90	14	153	60.3	54	110	14.8	48.46	15.30	31.57	
	Mean ± SD	89.00 ± 1.00	13.67 ± 0.58	152.00 ± 0.27	58.13 ± 2.37	54.00 ± 3.00	111.00 ± 4.58	15.13 ± 0.35	52.13 ± 3.56	15.33 ± 0.98	31.35 ± 0.27	
13	33-1	88	14	152	59.3	47	84	15.3	42.30	13.10	30.97	
14	33-2	86	13	154	51.2	41	76	15.7	38.75	12.65	32.65	
15	33-3	86	15	152	55.4	44	78	15.8	37.20	12.90	34.68	
	Mean ± SD	86.67 ± 1.53	14.00 ± 1.00	152.67 ± 1.15	55.30 ± 4.05	44.00 ± 3.00	79.33 ± 4.16	15.60 ± 0.26	39.42 ± 2.61	12.88 ± 0.22	32.77 ± 1.86	
16	39-1	85	14	152	57.4	51	97	15.8	47.40	15.11	31.88	
17	39-2	87	16	151	52.3	59	112	14.5	50.22	16.35	32.56	
18	39-3	88	16	154	51.6	55	102	15.4	47.45	15.76	33.21	
	Mean ± SD	86.67 ± 1.53	15.33 ± 1.15	152.33 ± 1.53	53.76 ± 3.16	55.00 ± 4.00	103.67 ± 7.64	15.23 ± 0.66	48.36 ± 1.61	15.74 ± 0.62	32.55 ± 0.66	
19	44-1	88	14	151	59.3	51	96	15.8	44.50	14.78	33.21	
20	44-2	85	13	151	55.3	58	110	14.7	46.54	15.40	33.09	
21	44-3	86	15	150	56.5	52	102	15.2	44.56	14.34	32.18	
	Mean ± SD	86.33 ± 1.53	14.00 ± 1.00	150.67 ± 0.58	57.03 ± 2.05	53.67 ± 3.78	102.67 ± 7.02	15.23 ± 0.55	45.20 ± 1.16	14.84 ± 0.53	32.83 ± 0.56	
22	BS 100E-1	81	9	147	49.4	17	29	13.6	24.55	4.55	18.53	
23	BS 100E-2	83	7	146	45.3	13	22	12.9	22.14	4.12	18.61	
24	BS 100E-3	83	9	148	44.8	14	25	13.7	21.45	4.22	19.67	
	Mean ± SD	82.33 ± 1.15	8.33 ± 1.15	147.00 ± 1.00	46.50 ± 2.52	14.67 ± 2.08	25.33 ± 3.51	13.40 ± 0.43	22.71 ± 1.63	4.29 ± 0.22	18.94 ± 0.64	
25	PBG7-1	88	16	153	59.3	58	114	15.9	51.44	16.5	32.08	
26	PBG7-2	89	17	154	54.5	54	107	16.2	47.62	15.4	32.34	
27	PBG7-3	88	16	155	57.6	52	100	15.7	49.55	15.8	31.89	
	Mean ± SD	88.33 ± 0.58	16.33 ± 0.58	154.00 ± 1.00	57.13 ± 2.43	54.67 ± 3.05	107.00 ± 7.00	15.93 ± 0.25	49.54 ± 1.91	15.9 ± 0.56	32.10 ± 0.22	

Data on BC_2F_2 population are presented for plants analyzed for recurrent parent genome recovery; data on BC_2F_3 population are based on three plants phenotypically similar to PBG7 and presented as mean ± SD; figures in parentheses are recurrent parent recovery percentages for agronomic traits calculated as plant trait value/value of PBG 7 for that trait × 100; [€] Harvest Index = Seed yield per plant/Biological yield × 100.
